# Catalytic Synthesis of a New Series of Alkyl Uronates and Evaluation of Their Physicochemical Properties

**DOI:** 10.3390/molecules21101301

**Published:** 2016-09-28

**Authors:** Huiling Lu, Audrey Drelich, Mehdi Omri, Isabelle Pezron, Anne Wadouachi, Gwladys Pourceau

**Affiliations:** 1 TIMR EA 4297 UTC/ESCOM, Sorbonne University, University of Technology of Compiègne, Rue Personne de Roberval, Compiègne CEDEX 60200, France; huiling.lu@utc.fr (H.L.); audrey.drelich@utc.fr (A.D.); isabelle.pezron@utc.fr (I.P.); 2 LG2A, UMR CNRS 7378—Chemistry Institute of Picardy FR CNRS 3085, University of Picardy Jules Verne, 33 rue Saint Leu, Amiens CEDEX 80039, France; mehdi.omri@u-picardie.fr (M.O.); anne.wadouachi@u-picardie.fr (A.W.)

**Keywords:** carbohydrate-based surfactants, uronic acids derivatives, sugar esters, TEMPO mediated oxidation

## Abstract

Large quantities (>3 g) of a new series of alkyl uronates were synthesized in two steps from commercial methyl hexopyranosides. Firstly, several tens of grams of free methyl α-d-glucopyranoside were selectively and quantitatively oxidized into corresponding sodium uronate using 2,2,6,6-tetramethyl-1-piperidinyloxy free radical (TEMPO)-catalyzed oxidation. Hydrophobic chains of different length were then introduced by acid-mediated esterification with fatty alcohols (ethyl to lauryl alcohol) leading to the desired alkyl glucuronates with moderate to good yields (49%–72%). The methodology was successfully applied to methyl α-d-mannopyranoside and methyl β-d-galactopyranoside. Physicochemical properties, such as critical micelle concentration (CMC), equilibrium surface tension at CMC (γ_cmc_), solubility, and Krafft temperature were measured, and the effect of structural modifications on surface active properties and micelle formation was discussed.

## 1. Introduction

Carbohydrate-based surfactants, including sugar esters, have recently become more and more attractive because of their renewable origin, biodegradability, non-toxicity, and physicochemical behaviors [1,2,3]. Among others properties, they are able to reduce the surface tension and to penetrate into lipid bilayers, are highly dispersible, and possess a high emulsifying power [4]. Moreover, some sugar-based surfactants exhibit antimicrobial activities [5]. All of these properties confer on them various applications in food, cosmetic, and pharmaceutical industries [6]. Sugar fatty esters are formed by one or several hydrophilic sugar headgroups and one or several hydrophobic alkyl chains. Due to their biodegradability, they constitute viable eco-friendly alternatives to petroleum-based surfactants [7]. The raw material (saccharides and fatty alcohols or acids) is available from renewable resources and obtained by simple processes: hydrolysis of starch, cellulose, hemicellulose, or pectin furnishes saccharides; steam-splitting of oils furnishes fatty chains. Moreover, the ester linkage between hydrophobic and hydrophilic parts is obtained by a simple esterification or acylation reaction performed by chemical or enzymatic synthetic routes. In the last decades, the interfacial properties of several series of sugar fatty esters have been investigated [8,9,10,11]. However, in most studies, anomeric-free carbohydrates are used, leading to mixtures of α and β isomers, for which the ratio was not determined. Yet, the configuration α or β of the sugar headgroup influences the micellar properties [12,13]. Thus, if one wants to establish the structure/function relationship between the molecular structure and the physicochemical properties of these sugar esters, it is essential to know exactly the α/β ratio or to protect the anomeric position. Moreover, there is still a need to design new sugar-based surfactants with better properties while understanding the impact of some structural modifications on the physicochemical properties. In particular, the ester “direction” (-C(O)-O-R, or -O-C(O)-R) can play a role on the air-water interface absorption [14]. Furthermore, both the sugar headgroup and the alkyl chain can affect the physicochemical properties of the glycolipids [15,16,17].

**Figure 1:**
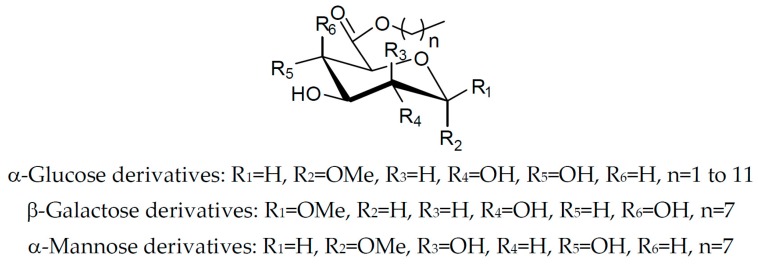
General structure of the proposed sugar fatty esters.

Herein, we propose to synthesize a new series of sugar fatty esters with well-defined structures (as described in Figure 1) and to evaluate their physicochemical properties.

With the aim to lead to viable eco-friendly alternatives to petroleum-based surfactants, the synthesis should be rapid, easy, scalable, and should respect green chemistry principles. The synthetic methodology should also be versatile and applicable to different headgroups and tail lengths. In this study, we synthesized, at the multigram scale, the proposed alkyl 1-methyl-uronates by, firstly, 2,2,6,6-tetramethyl-1-piperidinyloxy free radical (TEMPO)-catalyzed oxidation of methyl glycosides at primary position, followed by a H_2_SO_4_-catalyzed esterification with fatty alcohols. 

In order to measure the influence of the headgroup on the amphiphilic properties of this new series of sugar esters, the methodology was applied to different sugars (Man, Gal, Glc) with the same tail lengths as the C_8_ alkyl chain. The role of the hydrophobic tail length was also investigated using incrementally-increased hydrophobic chain lengths (C_6_, C_8_, C_10_, and C_12_ fatty alcohols) with an identical headgroup of Glc. The effect of the β configuration was studied for one molecule, via the octyl (1-β-methyl)galacturonate.

It is well known that solubility and surface adsorption properties are critically important aspects for most amphiphilic molecules. These properties, as well as phase diagram, are always influenced by the sugar head for sugar-based surfactants [18]. Amphiphilic molecules are rather convenient for industrial applications if they can dissolve easily at room temperature to a great degree, significantly reduce the surface tension, and have a large area of a soluble micellar phase. The present study is especially interested in the characterization of molecules having a C_6_, C_8_, C_10_, and C_12_ alkyl chain, the case for commonly ionic/nonionic used surfactants. Their water solubility and adsorption behavior, including Krafft point, concentration limit of clear solution, surface-tension lowering, and critical micellar concentration in water were studied to explore the influence of some structural factors, i.e., alkyl chain and sugar head.

## 2. Results and Discussion

### 2.1. Synthesis

Due to the electron-withdrawing effect of the C-5 carboxylic group that deactivates the anomeric position, functionalization of uronic acids at the anomeric position is particularly difficult, needing protection/deprotection steps and/or harsh conditions [19]. From our point of view, the best way to obtain the desired alkyl uronates respecting the atom-energy principle, and without the use of hazardous reactants/solvents, was to use a two-step protocol starting from commercial methyl glycoside: an oxidation step followed by an esterification reaction. Selective oxidation of primary alcohols remains challenging in carbohydrate chemistry when free carbohydrates are used. Whereas mild oxidants, such as Swern reagent or Dess-Martin periodinane, have been developed, regioselective oxidation of the primary hydroxyl group in the presence of the secondary ones is difficult. Therefore, the application of these reagents to polyols generally requires the use of protecting groups and increases the number of synthesis steps. As an environmentally benign and highly-regioselective catalyst, TEMPO and its derivatives, have been well described in the literature since the first report by Lebedev [20]. TEMPO is a stable radical able to oxidize alcohol into aldehyde and/or carboxylic acids in catalytic conditions, but needs to be regenerated in situ by co-catalysts. Various co-oxidants have been explored: NaOCl/KBr (Anelli′s procedure) [21], NaOCl/NaClO_2_ (Zhao′s procedure) [22], PhI(OAc)_2_ (also known as BAIB: iodobenzene diacetate) (Epp′s procedure) among others [23]. Avoiding the introduction/removal of inorganic salts is the major advantage of this latter method. Moreover, Epp′s procedure is especially suitable for large-scale reactions and the by-products are the rather innocuous iodobenzene and acetic acid. This was the procedure we chose for the oxidation of several grams of methyl glycoside.

**Scheme 1:**
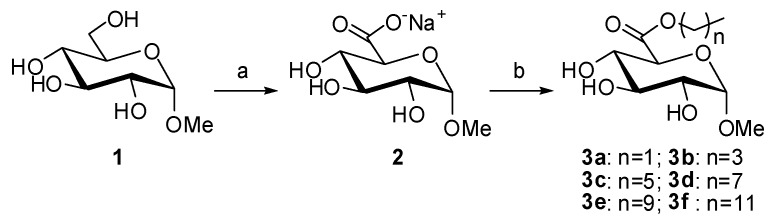
General procedure applied to methyl α-glucopyranoside. *Reagents and conditions*: (**a**) TEMPO cat., BAIB, NaHCO_3_, H_2_O/CH_3_CN, 15 h, RT; and (**b**) ROH, Na_2_SO_4_, H_2_SO_4_ cat., 24 h, 65 °C.

**Figure 2:**
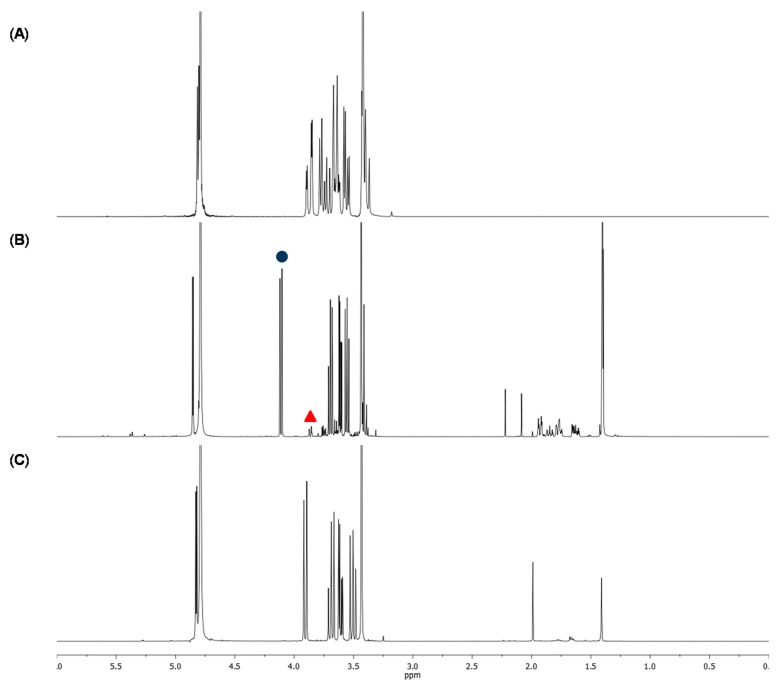
^1^H-NMR spectra of (**A**) methyl α-glucoside (starting material); (**B**) crude mixture without NaHCO_3_ after 17 days, (**C**) crude mixture with 1 eq. NaHCO_3_ after 24 h. Comparison between H-5 signal of α-methylglucuronic acid (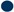) and H-6 of methyl α-glucoside (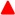) allows the evaluation of the conversion. From (**B**) to (**C**), the H-5 signal shifted from 4.1 to 3.9 ppm because of the basic pH (sodium carboxylate instead of carboxylic acid).

In order to establish the oxidation protocol (Scheme 1(a)), the reaction was first carried out by addition of TEMPO and BAIB to a solution of 1 g of methyl α-glucopyranoside in a water-acetonitrile solution. The reaction was monitored by TLC but, even after two weeks, incomplete oxidation was observed (~81% determined by ^1^H-NMR, Figure 2B). By performing the reaction in the presence of 1 eq. of NaHCO_3_, the reaction rate was drastically enhanced and the oxidation was complete within 24 h, as shown by ^1^H-NMR (Figure 2C). Indeed, alkaline conditions are known to favor TEMPO-catalyzed oxidations when NaOCl is used as co-oxidant. However, to our knowledge, in Epp′s procedure, no work never reported this particularity. In our case, the addition of NaHCO_3_ also led to a cleaner crude (almost no peak in the 1–2.5 ppm region). The resulting reaction mixture was quenched using ethanol and concentrated. Sodium (methyl α-d-glucopyranosid)uronate (**2**) was quantitatively obtained as white crystalline solid. These conditions were applied successfully to 5 g, then 15 g, of methyl α-glucopyranoside showing a good reproducibility on this substrate. When using methyl β-d-galactopyranoside and methyl α-D-mannopyranoside, although complete oxidations were obtained, the reaction rate was lower (48 h to 8 days were necessary). Considering the relatively clean NMR profiles obtained for the crudes and the difficulty to purify such polar molecules, the resultant crude sodium uronates were directly used for the following reaction, i.e., the esterification step.

Acid-catalyzed esterification of uronic acids is also challenging since, under acidic conditions, (trans)glycosylation and esterification are competitive, leading to several products [24]. Thus, a careful control of the esterification conditions is necessary to avoid by-product formation. Therefore, sodium methyluronates were used directly without exchange for uronic acids and the amount of H_2_SO_4_ was carefully controlled. As shown in Scheme 1(b), sodium 1-α-methylglucuronate was first dispersed in the desired alcohol under sonication, sodium sulfate was added as a desiccating agent, and the mixture was warmed to 65 °C. A catalytic amount of H_2_SO_4_ was then added. After 24 h, the reaction was quenched by the addition of Et_3_N, the residual alcohol was removed by evaporation or filtration on silica gel (depending on the boiling point of the alcohol), and the crude was purified by chromatography. Only traces of transglycosylated products were observed and the desired alkyl (1-α-methyl)glucuronates (**3a**–**3f**) were obtained in moderate to good yields (49%–72%).

**Figure 3:**
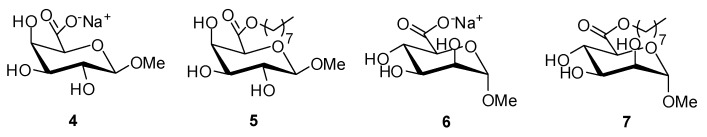
Molecular structures of sodium (1-β-methyl)galacturonate (**4**), octyl (1-β-methyl)galacturonate (**5**), sodium (1-α-methyl)mannuronate (**6**), and octyl (1-α-methyl)mannuronate (**7**).

To compare the effect of the sugar headgroup on the physicochemical properties, octyl (1-β-methyl)galacturonate (**5**) and octyl (1-α-methyl)mannuronate (**7**) were synthesized similarly starting from the corresponding sodium (1-β-methyl)galacturonate (**4**) and sodium (1-α-methyl)mannuronate (**6**), respectively (44%–57%) (Figure 3).

### 2.2. Aqueous Solubility

**Figure 4:**
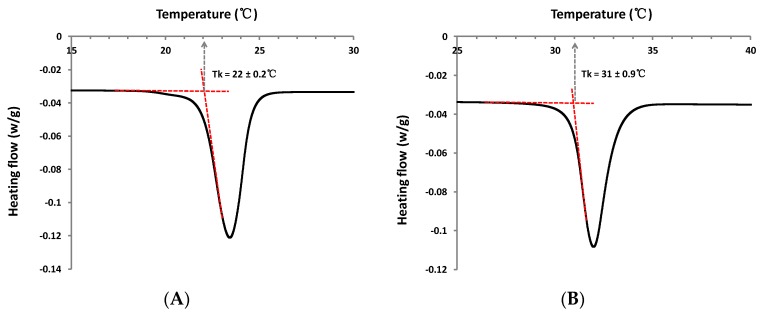
Representative DSC curves of phase transition for hydrated (**A**) dodecyl (1-α-methyl)glucuronate **3f** and (**B**) octyl (1-β-methyl)galacturonate **5**.

Results obtained from both visual observations and DSC (differential scanning calorimetry) analysis, for this new series of sugar esters, are summarized in Table 1. The appearance of the molecules is also reported. DSC analysis indicates that, solely, two molecules display a phase transition corresponding to a Tk (Krafft Temperature) in the temperature range studied. Corresponding typical DSC cooling curves of the phase transition obtained for these molecules in aqueous solution (10% *w*/*w*) are displayed in Figure 4. Moreover, the concentration limit of apparently clear solutions at room temperature of about 25 °C was pointed out for each molecule.

Firstly, the role of the hydrophobic tail length on the solubility of the molecules was investigated. For the alkyl (1-α-methyl)glucuronate series, with a gradual increase of hydrophobic chain length, hexyl and octyl (1-α-methyl)glucuronates (**3c** and **3d** respectively) appear more wax-like, while the decyl and dodecyl derivatives (**3e** and **3f** respectively) are both solid powders. Results suggest that as the chain length increases, the phase structure becomes more condensed. Regarding solubility, DSC results indicate that only dodecyl (1-α-methyl)glucuronate **3f** is characterized by a low Tk, at 22 ± 0.2 °C, near room temperature. DSC curves of other alkyl (1-α-methyl)glucuronates (**3c**–**3e**) are characterized by a straight line without any detected signal (not shown) between 4 °C and 65 °C. In addition, visual observations show that the concentration limit of apparently clear solutions for each molecule decreases as the chain length increases, from 50 mM for hexyl (1-α-methyl)glucuronate **3c** to 0.03 mM for the dodecyl derivative **3f**. It is important to note that the limit of limpid aqueous solutions have been observed at room temperature of about 25 °C, above the Tk of each alkyl (1-α-methyl)glucuronate studied here. Above this limit, solutions are turbid, and remain as such even when heating solutions up to 50 °C. A complete study of the phase diagram would be necessary to describe the evolution of the structures formed as a function of concentration and temperature. Abel et al. indicated that the solubility of sugar surfactants like dodecyl β-maltoside was defined by the stability of their crystal structures at a molecular level [25]. In accordance with this tendency, the results reveal that, for the alkyl (1-α-methyl)glucuronate series, the longer the alkyl chain is, the less soluble the molecule is, due to a more stable crystal structure (solid instead of wax).

Zhang et al. revealed that the nature of the sugar head plays an important role as to form a hydrogen bond network with water molecules to promote the dissolution of sugar-based surfactants in water [26]. In our study, for the octyl (1-α-methyl)uronates with variable sugar head, octyl (1-α-methyl)mannuronate **7** appears oily, contrary to the wax-like glucuronate derivative **3d**. Moreover, DSC curves of both molecules show no signal corresponding to the Tk detection. However, observations indicate that clear solutions of octyl (1-α-methyl)mannuronate **7** are obtained for concentrations up to 5 mM, slightly lower than for glucuronate derivative **3d** (20 mM). Concentrated solutions of compound **7** above this limit remain turbid even when heated. Considering an identical chain length, the mannose sugar head seems to have a more limited extent of water solubility in comparison to the glucose sugar head.

In the case of molecules in the β configuration, observations indicate that octyl (1-β-methyl)galacturonate **5** is also in a powder state and crystallized very easily during its purification process. DSC results reveal a Tk well above room temperature, at 31 ± 0.9 °C. Thus, observations show logically that this molecule, which is insoluble at room temperature (at about 25 °C, below Tk), is characterized by a large solubility (>100 mM) above 31 °C. In this case, the solubility increases considerably during the heating process. By applying a natural cooling of the concentrated solutions until room temperature, hydrated crystals are found to precipitate over time, and dispersions become clear again if they are reheated. A reversible solubilization-crystallization transition of octyl (1-β-methyl)galacturonate hydrated crystals is, therefore, observed. Compared to other alkyl α-uronates, due to the Krafft temperature of 31 °C, the solubility of the galacturonate derivative **5** is much more limited at room temperature. Above the Krafft point, the extent of aqueous solubility of compound **5** becomes much larger than other alkyl (1-α-methyl)uronates.

These various types of behavior were also observed for glucose-based surfactants by Boyd et al [27]. The authors indicated that, for high concentrations (above CMC) of alkyl polyglycosides, the concentration limit to obtain an apparently clear solution reflects a phase transition. Depending on the considered molecule, the boundary could correspond to the evolution from soluble micelles to other phase structures, and turbid solutions obtained could be constituted of insoluble phases, as hexagonal or lamellar dispersions, in equilibrium with or without micelles.

To sum up, the aqueous solubility of this new series of sugar esters depends on the alkyl chain length, the configuration, and the nature of the sugar head. It is important to note that these factors can either cooperate or compete with each other, which makes the aqueous solubility behavior more complicated and unpredictable for specific molecules.

### 2.3. Surface Activity

For each molecule, parameters related to the surface tension measurement are given in Table 2. The estimated standard deviations, regarding the graphic determination of the parameters, are also reported. 

**Figure 5:**
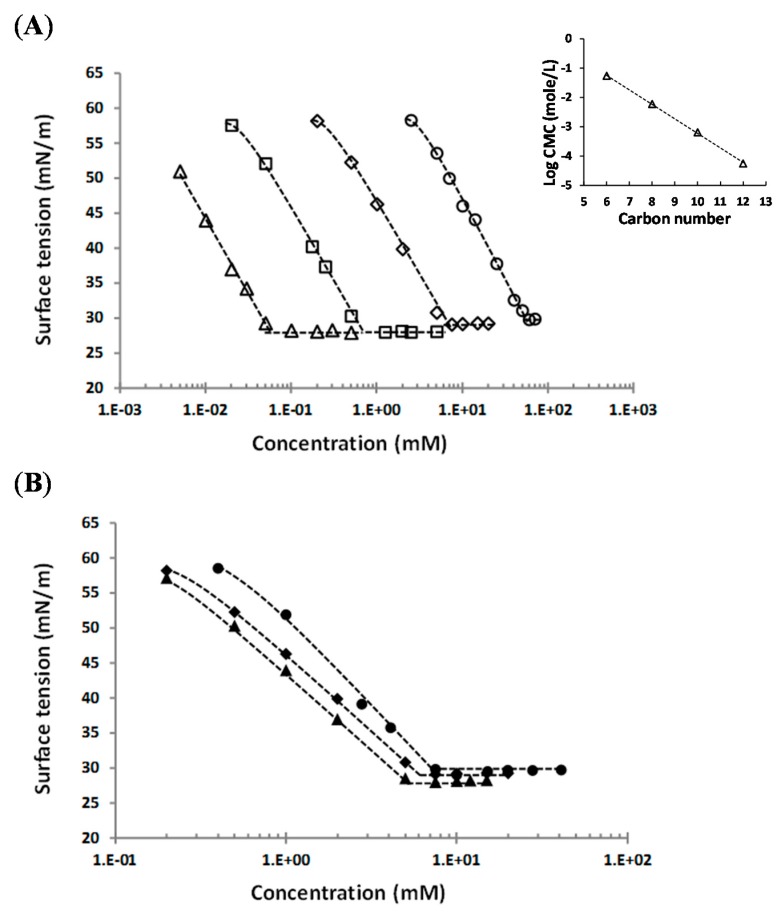
Representative curves of surface tension vs. molecule concentration for: (**A**) alkyl (1-α-methyl)glucuronate in water at 25 °C depending on chain length, ○—C_6_ (**3c**), ◇—C_8_ (**3d**), ☐—C_10_ (**3e**), △—C_12_ (**3f**), and (**B**) octyl esters derived from different sugars, ●—octyl (1-β-methyl)galacturonate **5** at 40 °C, ◆—octyl (1-α-methyl)glucuronate **3d** at 25 °C, ▲—octyl (1-α-methyl)mannuronate **7** at 25 °C. Insert: variation of Log CMC with the carbon numbers of the alkyl chain for alkyl (1-α-methyl)glucuronate at 25 °C. (----- guide line).

In all cases, the results reveal expected surfactant behavior, with a decrease of surface tension which becomes constant after a critical concentration is reached, i.e., the CMC. The corresponding curves of surface tension vs. concentration are displayed on Figure 5, at a controlled temperature of 25 °C, except for galacturonate derivative **5** at 50 °C, in accordance with the Tk value of 31 ± 0.9 °C. CMC, γ_cmc_, and A_min_ were deduced from these surface tension curves. The concentration limit of apparently clear solutions was also reported at 25 °C or 50 °C depending on Tk. In regards to calculated A_min_, results do not reveal a significant difference, A_min_ varying between 39.2 ± 2.9 Å^2^/molecule and 45.6 ± 5 Å^2^/molecule, whatever the alkyl chain length, the configuration, and the nature of the sugar head.

First, the alkyl chain lengh effect of alkyl (1-α-methyl)glucuronates on surface activity are discussed. Surface tension curves reveal that the CMC value decreases almost one decade with the increasing chain length of two carbons, from 55 ± 5 mM for the hexyl derivative **3c** to 0.056 ± 0.004 mM for the dodecyl derivative **3f**. Indeed, results show a linear relationship between the alkyl chain length and the Log CMC for alkyl (1-α-methyl)glucuronates at 25 °C (insert of Figure 5), which obeys the empirical equation used for surfactants [28,29,30]:

Log CMC= A − Bn
(1)

where n is the carbon numbers or the length of the alkyl chain; and A and B are empirical constants. The literature reported a rangeof values of B between 0.3 and 0.5 [28,29] with, generally, a value of B close to 0.3 for ionic surfactants, and close to 0.5 for non-ionic surfactants, according Rosen et al. [30]. For our synthesized alkyl α-glucuronates, the calculated values of A is 1.7 and B is 0.5, in agreement with the litterature for non-ionic surfactants.

In addition, results show a weak dependence of γ_cmc_ on the alkyl chain length: γ_cmc_ seems to slightly decrease when the number of carbon atoms increases, from 30.8 ± 0.8 mN/m for the hexyl α-glucuronate derivative **3c** to 28.3 ± 0.3 mN/m for the dodecyl α glucuronate derivative **3f**.

By comparing the surface activity of three octyl (1-methyl)uronates (α-glucuronate **3d**, β-galacturonate **5**, and α-mannuronate **7**), results indicate that values of CMC, γ_cmc_, and A_min_ are similar with the same order of magnitude: CMC is in the range 5.5–6.9 mM, γ_cmc_ is in the range 28.3–29.4 mN/m, and A_min_ is around 41–42.9 Å^2^/molecule. However, octyl (1-α-methyl)mannuronate **7** appears to be the most efficient with the lowest CMC (5.5 ± 0.5 mM) and γ_cmc_ (28.3 ± 0.3 mN/m) values, while octyl (1-β-methyl)galacturonate **5** is characterized by the highest CMC (6.9 ± 0.4 mM) and γ_cmc_ (29.4 ± 0.36 mN/m). Schmidt-Lassen et al. studied the CMC of pure sugar-based non-ionic surfactants and suggested that the CMC of octyl α-mannoside (10.9 mM) was comparable to the CMC of octyl α-glucoside, and smaller than the CMC of octyl β-galactoside (31.7 mM) [31]. Our results are in agreement with this tendency, and this series of octyl (1-methyl)uronates is characterized by lower CMC values than corresponding octyl glycosides. Moreover, the measured CMCs of the proposed molecules are much lower than the CMCs of most common ionic surfactants, like SDS (sodium dodecyl sulfate) (7.5 Mm–9.97 mM) [32]. Measured CMCs for decyl and dodecyl (1-α-methyl)glucuronates (**3e** and **3f**, respectively) are in the same order of magnitude than common polyoxyethylene (POE)-type non-ionic surfactants. For instance, the CMC was reported to be 0.03–0.09 mM for polyoxyethylene (23) lauryl ether (Brij 35), 0.23–0.24 mM for polyethylene glycol *tert*-octylphenyl ether (Triton X-100), and 0.050–0.059 mM for polyoxyethylene sorbitan monolaurate (Tween 20) [18,33,34,35].

In short, all of the proposed alkyl sugar esters, even for shorter alkyl chain (*n* = 6), demonstrate an excellent capability to reduce the surface tension of pure water to a great extent, in accordance with their amphiphilic structures. Results indicate that the surface activity depends mainly on the alkyl chain length and seems to be less influenced by the conformation of the sugar head.

By comparing the CMC values with the solubility limit [C]_max_ up to which a clear solution was observed, results reveal that only octyl β-galacturonate derivative **5** has a CMC value much lower than [C]_max_, highlighting a good property to form micelles, wanted for detergency applications and solubilization processes. In the case of alkyl (1-α-methyl)glucuronates, measurements indicate that only octyl derivative **3d** has a CMC value slightly lower than [C]_max_, while hexyl, decyl, and dodecyl derivatives (**3c**, **3e** and **3f** respectively) have a CMC in the range of [C]_max_ values, or CMC value slightly higher than [C]_max_, considering the standard deviation. In the same way, results show that octyl (1-α-methyl)mannuronate **7** has a CMC value in the range of [C]_max_ values. This behavior implies that, above [C]_max_, multiple non-miscible phases, including soluble micelles or not, and dispersion of structures, such as hexagonal or lamellar phases, could co-exist [27]. These results suggest that the observed change of slope in the surface tension curve may reveal a phase transition rather than a CMC. Direct methods, like solubilization of hydrophobic dyes detected by colorimetric analysis and small-angle scattering or dynamic light scattering experiments, should be used to evidence the presence of the micellar phase and confirm the CMC values. Therefore, this series of sugar esters appears to have a complicated phase diagram. As mentioned before, this complex solubility behavior was also observed for alkylpolyglucoside surfactants by Boyd et al. [27] and more recently on sugar-based surfactants with an alkyl or acyl chain grafted to a glucose or maltose head group by Lu et al. [36]. Obviously, a complete phase diagram should be investigated in further studies to understand and characterize the dissolution behavior and the solubility limit of each molecule. 

To sum up, except for octyl (1-α-methyl)glucuronate **3d** and octyl (1-β-methyl)galacturonate **5**, these results suggest a moderate ability of these alkyl sugar esters to form micelles, due to an area of limpid solution restricted and close to the CMC.

## 3. Materials and Methods

### 3.1. General Methods

All chemicals were purchased from Fisher Scientific (Illkirch, France), and Sigma Aldrich (St Quentin Fallavier, France) and used as received. Optical rotations were measured with a Perkin-Elmer Model 343 polarimeter (Perkin-Elmer Instruments, Courtaboeuf, France) using a sodium lamp at 20 °C. Optical rotation ([α]_D_) values are given in 10^−1^ deg cm^2^ g^−1^ and concentrations (c) are given in g for 100 mL. Mass analyses were performed on a Waters spectrometer (SINAPT TG2SI, Manchester, UK) using electrospray ionization (Z-Spray). NMR analysis were performed on a BRUKER DRX spectrometer (Bruker, Wissembourg, France) operating at 600 MHz for proton ^1^H and 151 MHz for ^13^C atoms or a spectrometer Bruker (Bruker) operating at 400 MHz for ^1^H and 100 MHz for ^13^C. Samples of oxidized products were prepared in deuterium oxide while samples of esters were dissolved in MeOD-*d*_4_.

### 3.2. General Procedure for the Oxidation

Methyl glycoside (methyl α-d-glucopyranoside, methyl β-d-galactopyranoside, or methyl α-d-mannopyranoside) (4–15 g) was firstly dissolved in deionized water (10 mL/g) and acetonitrile (10 mL/g) was added. TEMPO (0.3 eq.), BAIB (2.2 eq.), and NaHCO_3_ (1 eq.) were subsequently added and the mixture was stirred at 0 °C for 2 h and at room temperature. The reaction was monitored by TLC. After completion, the reaction was quenched by the addition of ethanol, followed by evaporation and decantation with H_2_O/EtOAc. The aqueous phase was concentrated and the resultant syrup was evaporated to dryness overnight to quantitatively give the corresponding sodium uronate as a white crystalline solid. For methyl α-d-glucopyranoside, the reaction was finished after 15h; for the methyl β-d-galactopyranoside, a second addition of TEMPO (0.3 eq.) and BAIB (2.2 eq.) was necessary and the reaction was finished after 48 h; for methyl α-d-mannopyranoside, two supplementary additions of TEMPO (0.3 eq.) and BAIB (2.2 eq.) and 8 days were necessary to oxidize all of the mannoside.

### 3.3. General Procedure for the Esterification

Sodium 1-methyluronate (5 g) was added to the desired fatty alcohol (100 mL) and a suspension was obtained. Sodium sulfate (5 g) and a catalytic amount of concentrated H_2_SO_4_ (0.9 mL) were successively added and the mixture was stirred at 65 °C. The reaction was monitored by TLC. After completion (~24 h), the mixture was neutralized with triethylamine until pH ~ 7, filtered, and evaporated. The crude was purified by flash column chromatography to give the corresponding ester. When using octanol, decanol, and dodecanol, the excess of alcohol was firstly removed using cyclohexane, then CH_2_Cl_2_/MeOH eluent (from 100/0 to 93/7) was used. 

### 3.4. Compound Characterization Data

All the NMR spectra are available in the Supplementary Materials.

*Sodium (methyl α-d-glucopyranosid)uronate* **2**. White powder. Quantitative yield from methyl α-d-glucopyranoside. ${\left[\alpha \right]}_{D}^{20}=+103{}^{\circ}$ (*c* 0.1; H_2_O); ^1^H-NMR (400 MHz, D_2_O) δ 4.83 (1H, d, H-1, *J*_1,2_ = 3.7 Hz), 3.90 (1H, d, H-5, *J*_4,5_ = 10.0 Hz), 3.69 (1H, dd, H-3, *J*_2,3_ = 9.8 Hz, *J*_3,4_ = 8.9 Hz), 3.61 (1H, dd, H-2, *J*_2,3_ = 9.7 Hz, *J*_1,2_ = 3.7 Hz), 3.51 (1H, dd, H-4, *J*_4,5_ = 10.0 Hz, *J*_3,4_ = 8.9 Hz), 3.43 (3H, s, -O-CH_3_). ^13^C-NMR (101 MHz, D_2_O) δ 176.6 (C=O), 99.3 (C-1), 72.8 (C-3), 71.9 (C-4, C-5), 71.0 (C-2), 55.1 (-O-CH_3_). HRMS (ESI) *m*/*z* calcd for C_7_H_12_O_7_ Na [M − Na]^+^ 231.0481, found 231.0489.

*Ethyl (methyl α-d-glucopyranosid)uronate* **3a**. Colorless syrup. Yield 58% from sodium (methyl α-d-glucopyranosid)uronate using the general procedure. *R*_f_ = 0.21 (CH_2_Cl_2_/MeOH = 93:7); ${\left[\alpha \right]}_{D}^{20}=+137{}^{\circ}$ (*c* 0.1; MeOH); ^1^H-NMR (400 MHz, MeOD-*d*_4_) δ 4.70 (1H, d, H-1, *J*_1,2_ = 3.7 Hz), 4.22 (2H, q, -O-CH_2_**-**, *J* = 7.1 Hz), 4.00 (1H, d, H-5, *J*_4,5_ = 9.8 Hz), 3.61 (1H, t, H-3, *J*_2,3_ = *J*_3,4_ = 9.2 Hz), 3.52 (1H, dd, H-4, *J*_4,5_ = 9.8 Hz, *J*_3,4_ = 9.2 Hz), 3.43 (4H, m, H-2,-O-CH_3_), 1.29 (3H, t, -CH_2_-CH_3_, *J* = 7.1 Hz). ^13^C-NMR (101 MHz, MeOD-*d*_4_) δ 171.5 (C=O), 101.9 (C-1), 74.5 (C-3), 73.3 (C-2), 73.1 (C-4), 72.9 (C-5), 62.5 (-O-CH_2_-), 56.0 (-O-CH_3_), 14.4 (-CH_3_). HRMS (ESI) *m*/*z* cald for C_9_H_16_O_7_Na [M + Na]^+^ 259.0794, found 259.0798.

*Butyl (methyl α-d-glucopyranosid)uronate* **3b**. White powder. Yield 72% from sodium (methyl α-d-glucopyranosid)uronate using the general procedure. *R*_f_ = 0.28 (EtOAc); ${\left[\alpha \right]}_{D}^{20}=+121{}^{\circ}$ (*c* 0.1; MeOH); ^1^H-NMR (400 MHz, MeOD-*d*_4_) δ 4.71 (1H, d, H-1, *J*_1,2_ = 3.7 Hz), 4.19 (2H, t, -O-CH_2_-, *J* = 6.6 Hz), 4.01 (1H, dd, H-5, *J*_4,5_ = 9.8 Hz, *J* = 0.4 Hz), 3.62 (1H, t, H-3, *J*_2,3_ = *J*_3,4_ = 9.2 Hz), 3.53 (1H, dd, H-4, *J*_4,5_ = 9.8 Hz, *J*_3,4_ = 9.2 Hz), 3.42 (4H, m, H-2,-O-CH_3_), 1.66 (2H, m, -O-CH_2_-CH_2_-), 1.42 (2H, m, -CH_2_-CH_3_), 0.96 (3H, t, -CH_2_-CH_3_, *J* = 7.4 Hz). ^13^C-NMR (101 MHz, MeOD-*d*_4_) δ 171.6 (C=O), 101.9 (C-1), 74.6 (C-3), 73.3 (C-2), 73.1 (C-4), 72.9 (C-5), 66.2 (-O-CH_2_-), 56.0 (-O-CH_3_), 31.7 (-O-CH_2_-CH_2_-), 20.1 (-CH_2_-CH_3_), 14.0 (-CH_3_). HRMS (ESI) *m*/*z* cald for C_11_H_20_O_7_Na [M + Na]^+^ 287.1107, found 287.1118.

*Hexyl (methyl α-D-glucopyranosid)uronate* **3c**. Colorless syrup. Yield 59% from sodium (methyl α-d-glucopyranosid)uronate using the general procedure. *R*_f_ = 0.23 (CH_2_Cl_2_/MeOH = 93:7); ${\left[\alpha \right]}_{D}^{20}=+103{}^{\circ}$ (*c* 0.1; MeOH); ^1^H-NMR (400 MHz, MeOD-*d*_4_) δ 4.71 (1H, d, H-1, *J*_1,2_ = 3.7 Hz), 4.18 (2H, t, -O-CH_2_**-**, *J* = 6.6 Hz), 4.01 (1H, d, H-5, *J*_4,5_ = 9.8 Hz), 3.62 (1H, t, H-3, *J*_2,3_ = *J*_3,4_ = 9.2 Hz), 3.53 (1H, dd, H-4, *J*_4,5_ = 9.8 Hz, *J*_3,4_ = 9.2 Hz), 3.45 (4H, m, H-2, -O-CH_3_), 1.68 (2H, p, -O-CH_2_-CH_2_**-**, *J* = 6.7 Hz), 1.36 (6H, m, -(CH_2_)_3_-CH_3_), 0.91 (3H, t, -CH_2_-CH_3_, *J* = 6.9 Hz). ^13^C-NMR (101 MHz, MeOD-*d*_4_) δ 171.6 (C=O), 101.9 (C-1), 74.6 (C-3), 73.3 (C-2), 73.1 (C-4), 72.9 (C-5), 66.5 (-O-CH_2_-), 56.0 (-O-CH_3_), 32.6, 29.5, 26.6, 23.6 (-O-CH_2_-(CH_2_)_4_-CH**_3_**), 14.3 (-CH_3_). HRMS (ESI) *m*/*z* cald for C_13_H_25_O_7_ [M + H]^+^ 293.1600, found 293.1608.

*Octyl (methyl α-D-glucopyranosid)uronate* **3d**. White syrup. Yield 57% from sodium (methyl α-d-glucopyranosid)uronate using the general procedure. *R*_f_ = 0.24 (CH_2_Cl_2_/MeOH = 93:7); ${\left[\alpha \right]}_{D}^{20}=+99{}^{\circ}$ (*c* 0.1; MeOH); ^1^H-NMR (400 MHz, MeOD-*d*_4_) δ 4.71 (1H, d, H-1, *J*_1,2_ = 3.7 Hz), 4.18 (2H, t, -O-CH_2_**-**, *J* = 6.6 Hz), 4.01 (1H, d, H-5, *J*_4,5_ = 9.8 Hz), 3.62 (1H, t, H-3, *J*_2,3_ = *J*_3,4_ = 9.2 Hz), 3.52 (1H, dd, H-4, *J*_4,5_ = 9.8 Hz, *J*_3,4_ = 9.2 Hz), 3.45 (4H, m, H-2, -O-CH_3_), 1.68 (2H, p, -O-CH_2_-CH_2_**-**, *J* = 6.6 Hz), 1.35 (10H, m, -(CH_2_)_5_-CH_3_), 0.91 (3H, t, -CH_2_-CH_3_, *J* = 6.8 Hz). ^13^C-NMR (101 MHz, MeOD-*d*_4_) δ 171.6 (C=O), 101.9 (C-1), 74.5 (C-3), 73.3 (C-2), 73.1 (C-4), 72.9 (C-5), 66.5 (-O-CH_2_-), 56.0 (-O-CH_3_), 33.0, 30.3, 30.3, 29.9, 26.9, 23.7 (-O-CH_2_-(CH_2_)_6_-CH**_3_**), 14.4 (-CH_3_). HRMS (ESI) *m*/*z* cald for C_15_H_28_O_7_Na [M + Na]^+^ 343.1733, found 343.1744.

*Decyl (methyl α-d-glucopyranosid)uronate* **3e**. White powder. Yield 63% from sodium (methyl α-d-glucopyranosid)uronate using the general procedure. *R*_f_ = 0.4 (EtOAc/MeOH = 95:5); ${\left[\alpha \right]}_{D}^{20}=+93{}^{\circ}$ (*c* 0.1; MeOH); ^1^H-NMR (400 MHz, MeOD-*d*_4_) δ 4.71 (1H, d, H-1, *J*_1,2_ = 3.7 Hz), 4.18 (2H, t, -O-CH_2_**-**, *J* = 6.6 Hz), 4.01 (1H, d, H-5, *J*_4,5_ = 9.8 Hz), 3.62 (1H, t, H-3, *J*_2,3_ = *J*_3,4_ = 9.2 Hz), 3.52 (1H, dd, H-4, *J*_4,5_ = 9.8 Hz, *J*_3,4_ = 9.2 Hz), 3.44 (4H, m, H-2, -O-CH_3_), 1.68 (2H, p, -O-CH_2_-CH_2_**-**, *J* = 6.6 Hz), 1.35 (14H, m, -(CH_2_)_7_-CH_3_), 0.90 (3H, t, -CH_2_-CH_3_, *J* = 6.8 Hz). ^13^C-NMR (101 MHz, MeOD-*d*_4_) δ 171.6 (C=O), 101.9 (C-1), 74.5 (C-3), 73.3 (C-2), 73.1 (C-4), 72.9 (C-5), 66.5 (-O-CH_2_-), 56.0 (-O-CH_3_), 33.1, 30.7 (2C), 30.5, 30.4, 29.6, 26.9, 23.7 (-O-CH_2_-(CH_2_)_8_-CH_3_), 14.5 (-CH_3_). HRMS (ESI) *m*/*z* cald C_17_H_36_NO_7_ [M + NH_4_]^+^ 366.2492, found 366.2499.

*Dodecyl (methyl α-d-glucopyranosid)uronate* **3f**. White powder. Yield 49% from sodium (methyl α-d-glucopyranosid)uronate using the general procedure. *R*_f_ = 0.35 (EtOAc); ${\left[\alpha \right]}_{D}^{20}=+82{}^{\circ}$ (*c* 0.1; MeOH); ^1^H-NMR (400 MHz, MeOD-*d*_4_) δ 4.71 (1H, d, H-1, *J*_1,2_ = 3.7 Hz), 4.18 (2H, t, -O-CH_2_-, *J* = 6.6 Hz), 4.01 (1H, d, H-5, *J*_4,5_ = 9.8 Hz), 3.62 (1H, t, H-3, *J*_2,3_ = *J*_3,4_ = 9.2 Hz), 3.52 (1H, dd, H-4, *J*_4,5_ = 9.8 Hz, *J*_3,4_ = 9.2 Hz), 3.44 (4H, m, H-2, -O-CH_3_), 1.68 (2H, p, -O-CH_2_-CH_2_**-**, *J* = 6.6 Hz), 1.32 (18H, m, -(CH_2_)_9_-CH_3_), 0.89 (3H, t, -CH_2_-CH_3_, *J* = 6.8 Hz). ^13^C-NMR (101 MHz, MeOD-*d*_4_) δ 171.6 (C=O), 101.9 (C-1), 74.6 (C-3), 73.3 (C-2), 73.1 (C-4), 72.9 (C-5), 66.5 (-O-CH_2_-), 56.0 (-O-CH_3_), 33.1, 30.8, 30.8, 30.7, 30.7, 30.5, 30.4, 29.6, 26.9, 23.8 (-O-CH_2_-(CH_2_)_8_-CH**_3_**), 14.5 (-CH_3_). HRMS (ESI) *m*/*z* cald C_19_H_36_O_7_Na [M + Na]^+^ 399.2359, found 399.2368.

*Sodium (methyl β-d-galactopyranosid)uronate* **4**. White powder. Quantitative yield from methyl β-d-galactopyranoside. ${\left[\alpha \right]}_{D}^{20}=-37{}^{\circ}$ (*c* 0.1; H_2_O); ^1^H-NMR (400 MHz, D_2_O) δ 4.27 (1H, d, H-1, *J*_1,2_ = 7.9 Hz), 4.18 (1H, dd, H-4, *J*_3,4_ = 3.5 Hz, *J*_4,5_ = 1.2 Hz), 4.01 (1H, d, H-5, *J*_4,5_ = 1.3 Hz), 3.66 (1H, dd, H-3, *J*_2,3_ = 9.9 Hz, *J*_3,4_ = 3.5 Hz), 3.56 (3H, s, -O-CH_3_), 3.48 (1H, dd, H-2, *J*_2,3_ = 9.9 Hz, *J*_1,2_ = 7.9 Hz). ^13^C-NMR (101 MHz, D_2_O) δ 175.0 (C=O), 103.2 (C-1), 75.4 (C-5), 72.8 (C-3), 70.3 (C-2), 70.3 (C-4), 57.1 (-O-CH_3_). HRMS (ESI) *m*/*z* calcd for C_7_H_12_O_7_Na [M + Na]^+^ 231.0481, found 231.0488.

*Octyl (methyl β-d-galactopyranosid)uronate* **5**. White powder. Yield 57% from sodium (methyl β-d-galactopyranosid)uronate using the general procedure. *R*_f_ = 0.29 (CH_2_Cl_2_/MeOH = 93:7); ${\left[\alpha \right]}_{D}^{20}=-33{}^{\circ}$ (*c* 0.1; MeOH); ^1^H-NMR (400 MHz, MeOD-*d*_4_) δ 4.26 (1H, d, H-5, *J*_4,5_ = 1.4 Hz), 4.18 (3H, m, H-1, -O-CH_2_-), 4.13 (1H, dd, H-4, *J*_4,5_ = 1.4 Hz, *J*_3,4_ = 2.9 Hz), 3.54 (5H, m, H-2, H-3, -O-CH_3_), 1.68 (2H, p, -O-CH_2_-CH_2_-, *J* = 6.7 Hz), 1.35 (10H, m, -(CH_2_)_5_-CH_3_), 0.9 (3H, m, -CH_2_-CH_3_, *J* = 6.9 Hz). ^13^C-NMR (101 MHz, MeOD-*d*_4_) δ 170.3 (C=O), 105.7 (C-1), 75.4 (C-5), 74.4 (C-3), 71.8 (C-2), 71.5 (C-4), 66.4 (-O-CH_2_-), 57.5 (-O-CH_3_), 33.0, 30.3 (2C), 29.7, 27.0, 23.7 (-O-CH_2_-(CH_2_)_6_-CH**_3_**), 14.4 (-CH_3_). HRMS (ESI) *m*/*z* cald for C_15_H_29_O_7_ [M + H]^+^ 321.1913, found 321.1898.

*Sodium (methyl α-d-mannopyranosid)uronate* **6**. White powder. Quantitative yield from methyl α-d-mannopyranoside. ${\left[\alpha \right]}_{D}^{20}=+40{}^{\circ}$ (*c* 1.0; H_2_O); ^1^H-NMR (400 MHz, D_2_O) δ 4.77 (1H, d, H-1, *J*_1,2_ = 1.9 Hz), 3.88 (1H, dd, H-2, *J*_1,2_ = 1.9 Hz, *J*_2,3_ = 3.0 Hz), 3.86–3.73 (3H, m, H-3, H-4, H-5), 3.39 (3H, s, -O-CH_3_). ^13^C-NMR (101 MHz, D_2_O) δ 176.7 (C=O), 100.8 (C-1), 72.8 (C-5), 70.3 (C-3), 69.7 (C-2), 68.7 (C-4), 54.9 (-O-CH_3_). HRMS (ESI) *m*/*z* calcd for C_7_H_12_O_7_ Na [M + Na]^+^ 231.0481, found 231.0487.

*Octyl (methyl α-d-mannopyranosid)uronate* **7**. Yellow syrup. Yield 44% from sodium (methyl α-d-mannopyranosid)uronate using the general procedure. *R*_f_ = 0.31 (CH_2_Cl_2_/MeOH = 93:7); ${\left[\alpha \right]}_{D}^{20}=+58{}^{\circ}$ (*c* 0.1; MeOH); ^1^H-NMR (400 MHz, MeOD-*d*_4_) δ 4.71 (1H, d, H-1, *J*_1,2_ = 2.2 Hz), 4.17 (2H, t, -O-CH_2_-, *J* = 6.6 Hz), 3.98 (1H, d, H-5, *J*_4,5_ = 9.0 Hz), 3.92 (1H, t, H-4, *J*_3,4_ = *J*_4,5_ = 8.9 Hz), 3.78 (1H, dd, H-2, *J*_1,2_ = 2.3 Hz, *J*_2,3_ = 3.3 Hz), 3.67 (1H, dd, H-3, *J*_2,3_ = 3.4 Hz, *J*_3,4_ = 8.6 Hz), 3.40 (3H, s, -O-CH_3_), 1.68 (2H, p, -O-CH_2_-CH_2_-, *J* = 6.6 Hz), 1.34 (m, 10H, -(CH_2_)_5_-CH_3_), 0.89 (m, 3H, -CH_2_-CH_3_, *J* = 6.9 Hz). ^13^C-NMR (101 MHz, MeOD-*d*_4_) δ 171.5 (C=O), 103.2 (C-1), 74.0 (C-5), 72.1 (C-3), 71.4 (C-2), 69.8 (C-4), 66.4 (-O-CH_2_-), 55.7 (-O-CH_3_), 32.9, 30.3 (2C), 29.6, 26.9, 23.7 (-O-CH_2_-(CH_2_)_6_-CH**_3_**), 14.4 (-CH_3_). HRMS (ESI) *m*/*z* cald for C_15_H_28_O_7_Na [M + Na]^+^ 343.1733, found 343.1746.

### 3.5. Determination of Aqueous Solubility 

Firstly, the appearance of pure, synthesized molecules and the solubility area of the synthesized molecules in water were appreciated by visual observation at room temperature of about 25 °C. For each molecule, several samples in 1–2 mL deionized water with various concentrations (between 0.01 mM and 100 mM) were prepared by homogenizing at 500 rpm with a standard magnetic hotplate stirrer MR 3001K (Heidolph Instruments, Schwabach, Germany) for 30 min. The soluble or insoluble character was estimated on the aspect change of corresponding dispersion systems. The concentration limit to obtain an apparently clear solution was noted [C]_max_. When solubilization does not occur at room temperature, a slow heating to 50 °C was applied under magnetic agitation. This allows the examination of the temperature-driven solubilization behavior and verifies the presence of the Krafft point (Tk), above which the surfactant solubility in water increases abruptly [37]. At Tk, solubility of the surfactant reaches its critical micelle concentration (CMC) [38]. In other words, Tk represents the critical temperature at which the micellization process occurs with the dissolution of hydrated surfactant crystals [39]. Thus, Tk can be precisely determined by differential scanning calorimetry (DSC), which has already been proposed by some authors [40,41]. All of the measurements were performed with a commercial µDSC7 evo calorimeter (SETARAM Instrumentation, Caluire, France). For each molecule, a sample with a concentrated aqueous mixture of 10% (*w*/*w*) molecule (C >> CMC) was prepared at 50 °C under magnetic agitation for 30 min to obtain a homogeneous and clear dispersion. These dispersions were then put into a refrigerator at 4 °C for a period over a week, permitting potential phase transitions, including the formation of equilibrated hydrated crystals. Samples of about 300 mg were introduced in the calorimeter cell and equilibrated at 4 °C for 1 h. A heating scan from 4 °C to 65 °C at a rate of 0.5 °C/min was then performed. Tk were identified as the onset of the endothermic signal detected by DSC during the dissolution of hydrated crystals. Tk were calculated as an average of at least two measurements.

### 3.6. Determination of the Surface Activity

The surface activity of the synthesized molecules was studied mainly in terms of their adsorption behavior on the air-aqueous solution interface. The equilibrium surface tensions γ were measured by means of a Wilhelmy plate using a K100 Processor Tensiometer (KRUSS, Hamburg, Germany). The equilibrium surface tension γ was plotted as a function of the log concentration for all molecules, from which we can derive various parameters, like critical micellar concentration (CMC) extrapolated from the break in the surface tension curve and surface tension at the CMC (γ_cmc_). The surface excess (Γ_max_) and minimal area per molecule at air-aqueous solution interface (A_min_) can be calculated from Gibbs equation for non-ionic surfactants:

(2) $${Г}_{\max}=-\frac{1}{2.303RT}\left(\frac{d\gamma }{dlogc}\right)$$

and

(3) $$A_{\min}=\frac{10^{20}}{N_{A}{Г}_{\max}}$$

where R is the ideal gas constant (8.31 J·mol^−1^K^−1^)’ T is the measuring temperature (K); γ is the surface tension (N·m^−1^), c is the concentration (mol·L^−1^); A_min_ is the minimal area per molecule (Å^2^); and N_A_ is Avogadro′s number (6.02 × 10^23^ mol^−1^).

For each molecule, a series of solutions of different concentrations were prepared well above its Krafft Point, either at room temperature of about 25 °C or at 50 °C, under agitation in a water bath for 30 min. Measurements were carried out for each concentration, either at 25 °C for molecules with a Tk below room temperature, or at T > Tk for those with higher Tk values than 25 °C. The standard deviation for temperature is 1 °C.

## 4. Conclusions

A new family of sugar-based surfactants was synthesized at the multigram scale using a simple and rapid two-step protocol from commercial methyl glycosides and characterized by physicochemical analyses. This methodology, which uses TEMPO-catalyzed oxidation and acid-catalyzed esterification, was applied to variable sugar heads (Glc, Gal, Man) and fatty alcohols (ethyl to lauryl alcohol) and led to the corresponding alkyl (1-methyl)uronates with moderate to good yields (44%–72% for two steps). The role of the chain length on the interfacial properties was evaluated at 25 °C using C_6_–C_12_ derivatives of alkyl (1-α-methyl)glucuronates. CMC values were shown to significantly decrease when the length of the chain increases, while γ_cmc_ slightly increases with the number of carbons of the alkyl chain. On the other hand, for a given alkyl chain length (C_8_), sugar head nature (Gal, Glc, or Man) does not influence the interfacial properties. So, as yet observed for others sugar-based surfactants, a small variation in the alkyl chain length has a greater effect on the surface activity than the nature of the sugar head. This study reveals that these sugar esters are of particular interest, resulting in a significant reduction of the effective amount of molecules required to give a minimum surface tension of pure water, similar to corresponding alkylpolyglycosides or common polyoxyethylene nonionic surfactants. However, the proposed molecules present a limited micellar phase area, whatever the alkyl chain length or the nature of the sugar head. Indeed, the solubility limits to obtain a clear solution significantly decrease when the length of the chain increases. This point can be problematic for industrial applications and can lead to a decrease in the ease of their handing and formulating. Nevertheless, this work illustrates that anticipating the solubility properties of new amphiphilic molecules is tricky and challenging. The improvement of molecular structures and synthesis of new sugar ester surfactants should be investigated to enhance sugar ester solubility properties and explore their potential applications in accordance with specific industrial needs. For example, additional results indicate that synthesized sugar esters with a galactose sugar head enhance the solubility.

## Figures and Tables

**Table 1:** Physical parameters related to solubility property for studied molecules.

Molecule	Tk ^a^ (°C)	[C]_max_ at ≈25 °C (mM)	State
Hexyl (1-α-methyl)glucuronate **3c**	undetected	50–60	wax
Octyl (1-α-methyl)glucuronate **3d**	undetected	15–20	wax
Decyl (1-α-methyl)glucuronate **3e**	undetected	0.5–0.75	powder
Dodecyl (1-α-methyl)glucuronate **3f**	22 ± 0.2	0.03–0.05	powder
Octyl (1-β-methyl)galacturonate **5**	31 ± 0.9	insoluble at ≈ 25 °C; >100 at T > Tk	powder
Octyl (1-α-methyl)mannuronate **7**	undetected	5.0–6.2	oil

^a^ Krafft Temperature.

**Table 2:** Physical parameters of surface activity for investigated molecules.

Molecule	CMC (mM)	[C]_max_ (mM)	γ_cmc_ (mN·m^−1^)	A_min_ (Å^2^/molecule)	T (°C)
Hexyl (1-α-methyl)glucuronate **3c**	55 ± 5	50–60	30.8 ± 0.8	39.2 ± 2.9	25
Octyl (1-α-methyl)glucuronate **3d**	6.0 ± 0.5	15–20	29.2 ± 0.4	41.0 ± 1	25
Decyl (1-α-methyl)glucuronate **3e**	0.65 ± 0.05	0.5–0.75	28.1 ± 0.1	42.4 ± 2	25
Dodecyl (1-α-methyl)glucuronate **3f**	0.056 ± 0.004	0.03–0.05	28.3 ± 0.3	45.6 ± 5	25
Octyl (1-β-methyl)galacturonate **5**	6.9 ± 0.4	>100	29.4 ± 0.4	42.9 ± 3.9	40
Octyl (1-α-methyl)mannuronate **7**	5.5 ± 0.5	5.0–6.2	28.3 ± 0.3	42.8 ± 2	25

## Supplementary Materials

The ^1^H- and ^13^C-NMR spectral of compounds **2**, **3a**–**3f**, **4**, **5**, **6** and **7** are summarized in Supplementary Materials. Supplementary materials can be accessed at: http://www.mdpi.com/1420-3049/21/10/1301/s1.
