# Supplementary material for: Catalytic Synthesis of a New Series of Alkyl Uronates and Evaluation of Their Physicochemical Properties
Source: Molecules. 2016 Sep 28;21(10):1301. doi: 10.3390/molecules21101301 (PMC6273592; doi:10.3390/molecules21101301)
Supplement: Supplementary file 1 [file molecules-21-01301-s001.pdf]

Supplementary Materials: Catalytic Synthesis of a New Series of Alkyl Uronates and Evaluation of Their Physicochemical Properties

Huiling Lu, Audrey Drelich, Mehdi Omri, Isabelle Pezron, Anne Wadouachi and Gwladys Pourceau

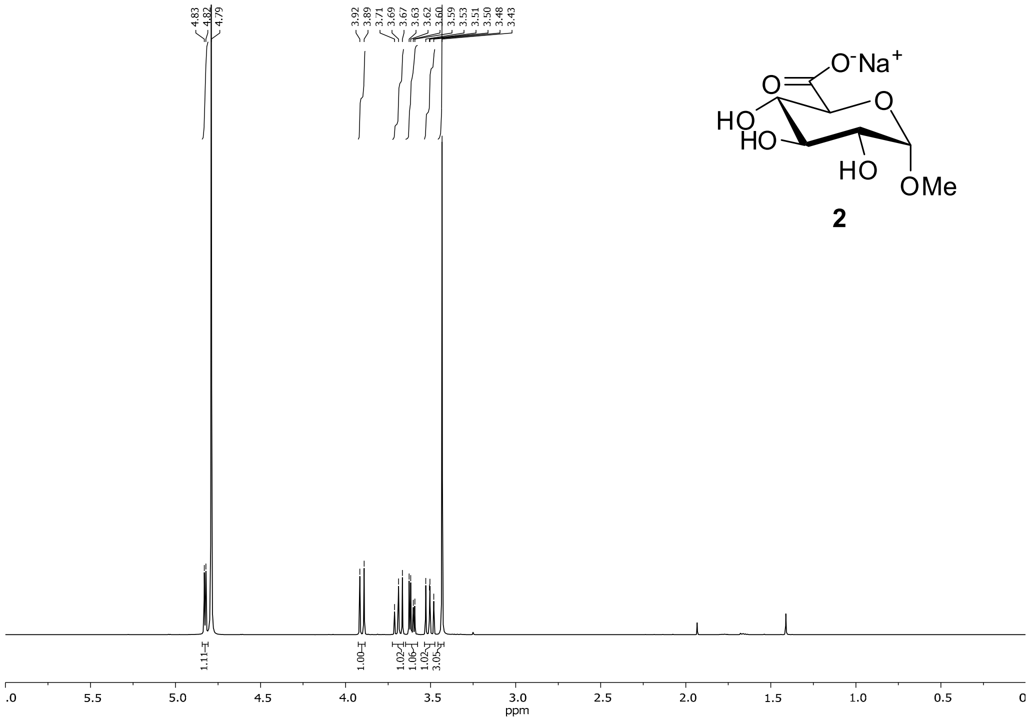

Figure S1. ^{1}H-NMR spectra of sodium (methyl α-D-glucopyranosid)uronate **2**.

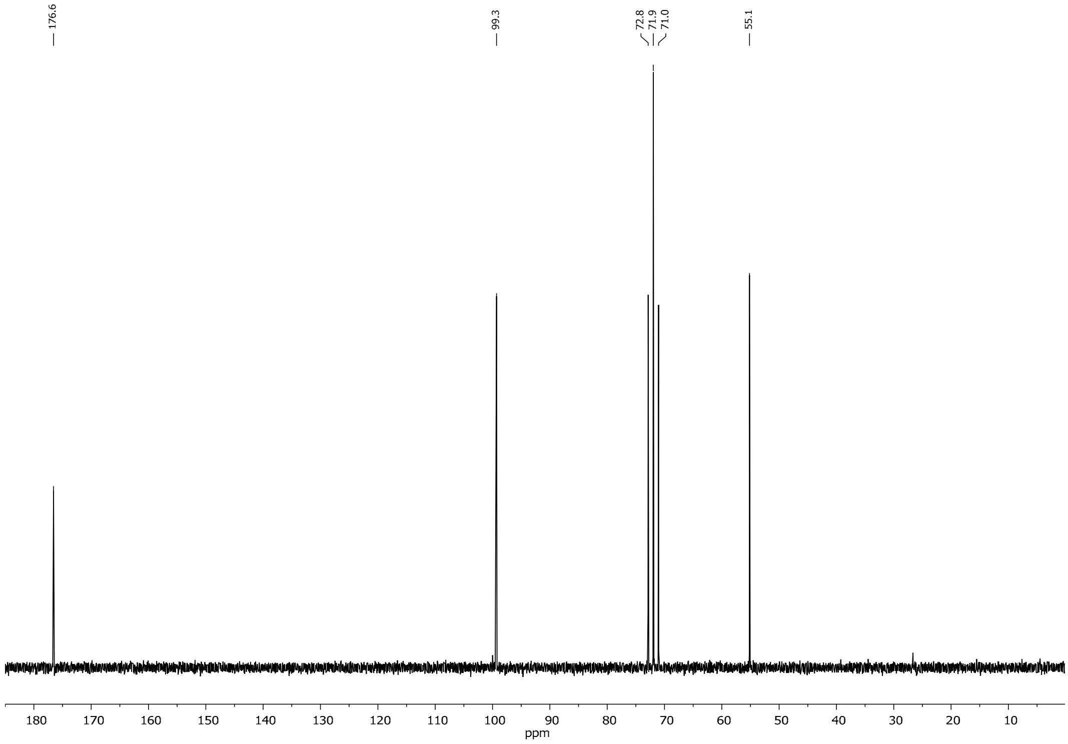

Figure S2. ^{13}C-NMR spectra of sodium (methyl α-D-glucopyranosid)uronate **2**.

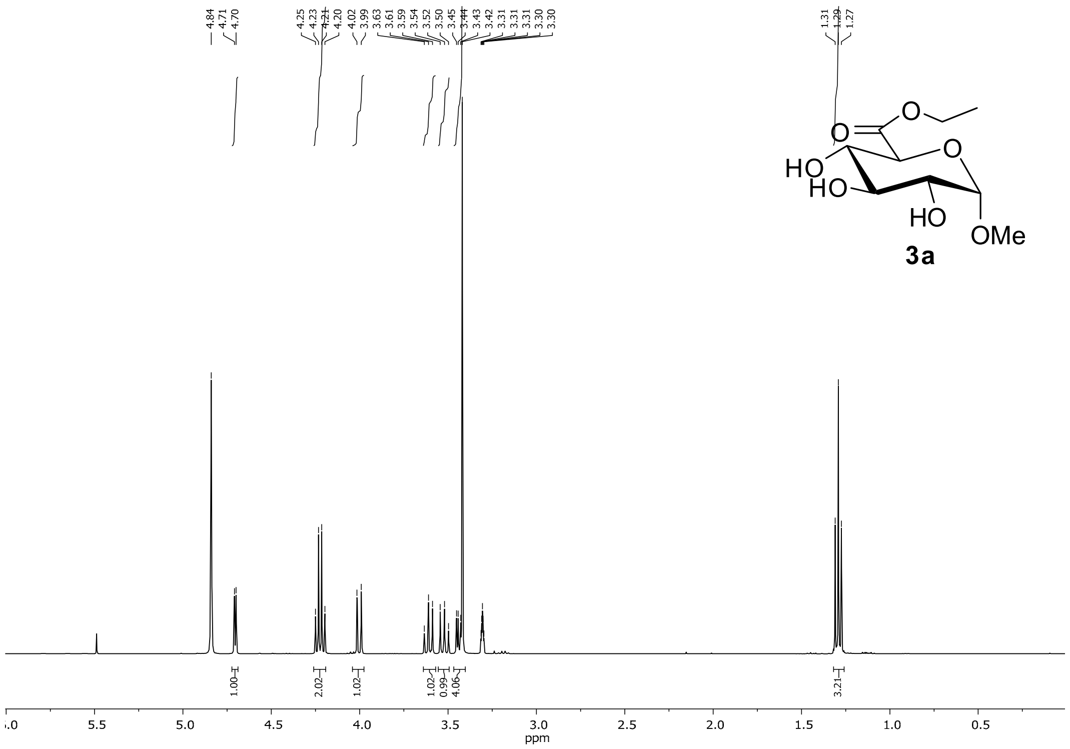

Figure S3. ^{1}H-NMR spectra of ethyl (methyl α-D-glucopyranosid)uronate **3a**.

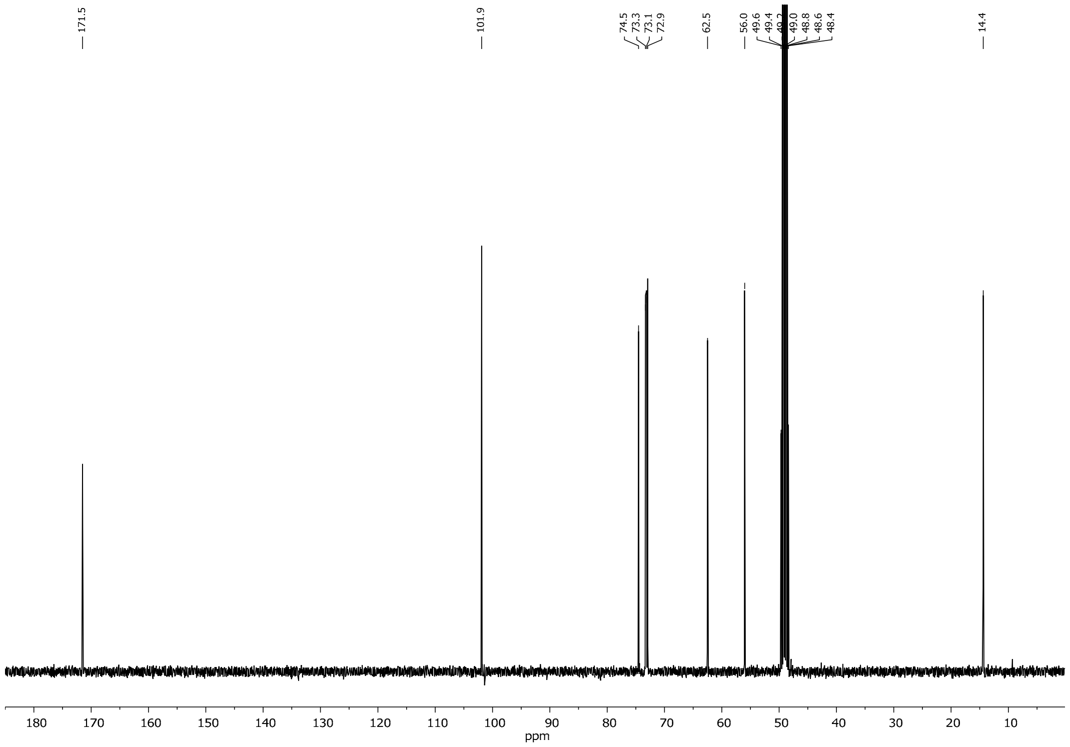

Figure S4. ^{13}C-NMR spectra of ethyl (methyl α-D-glucopyranosid)uronate **3a**.

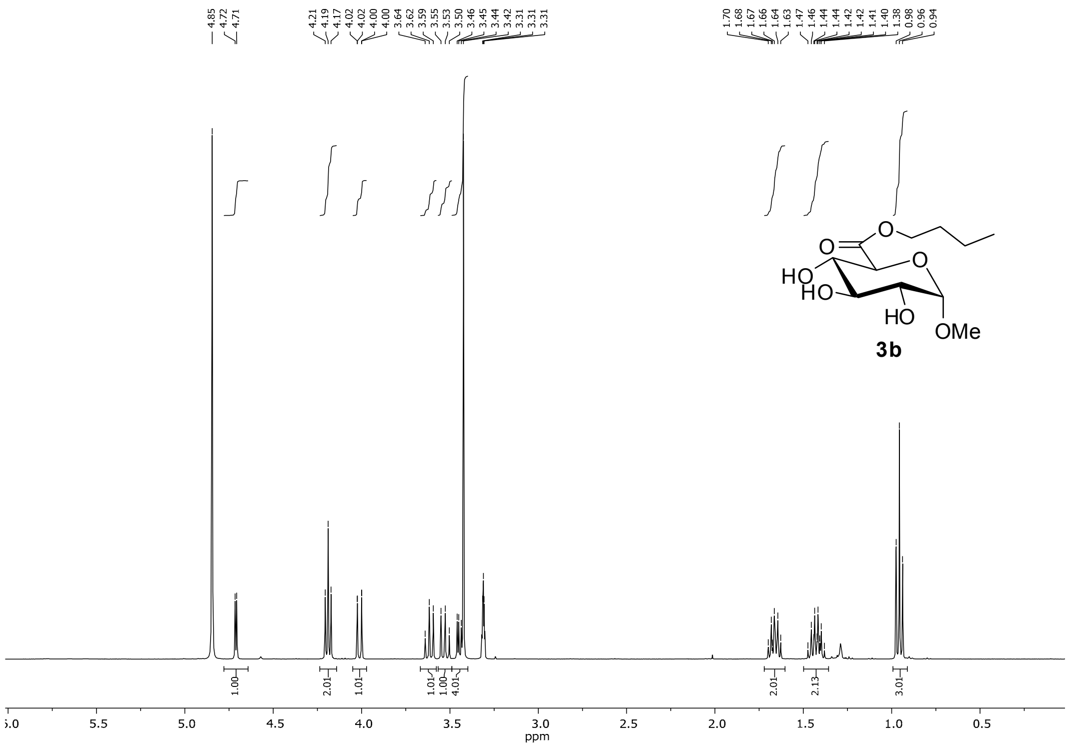

Figure S5. ^{1}H-NMR spectra of butyl (methyl *α*-D-glucopyranosid)uronate **3b**.

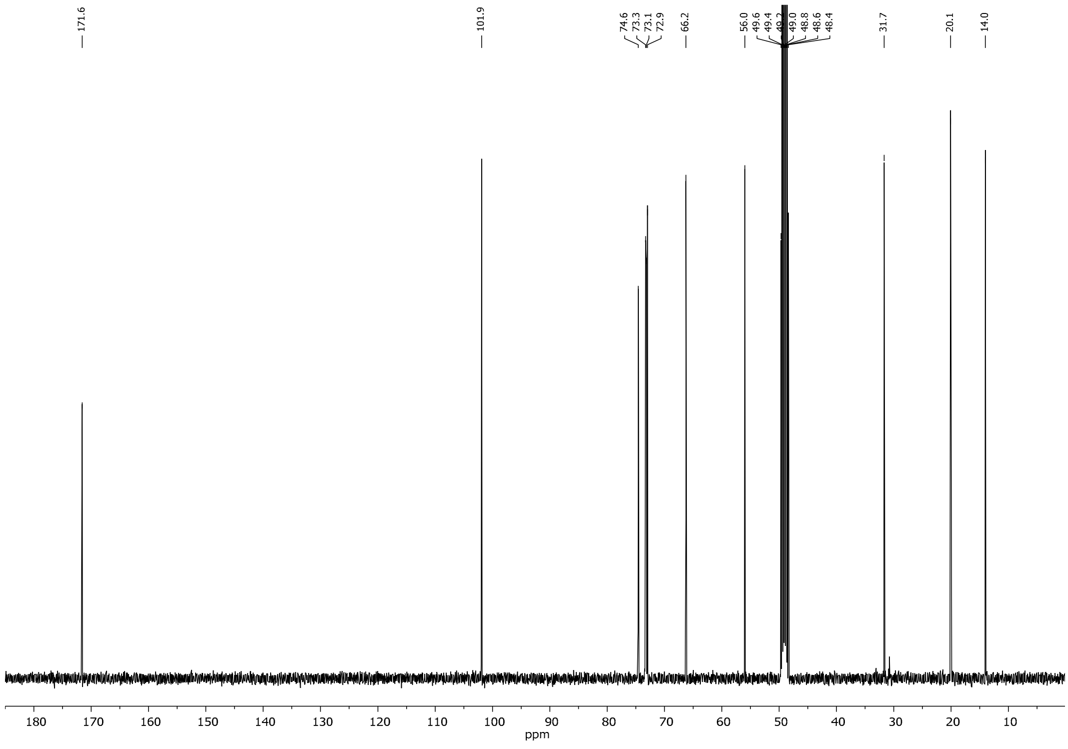

Figure S6. ^{13}C-NMR spectra of butyl (methyl *α*-D-glucopyranosid)uronate **3b**.

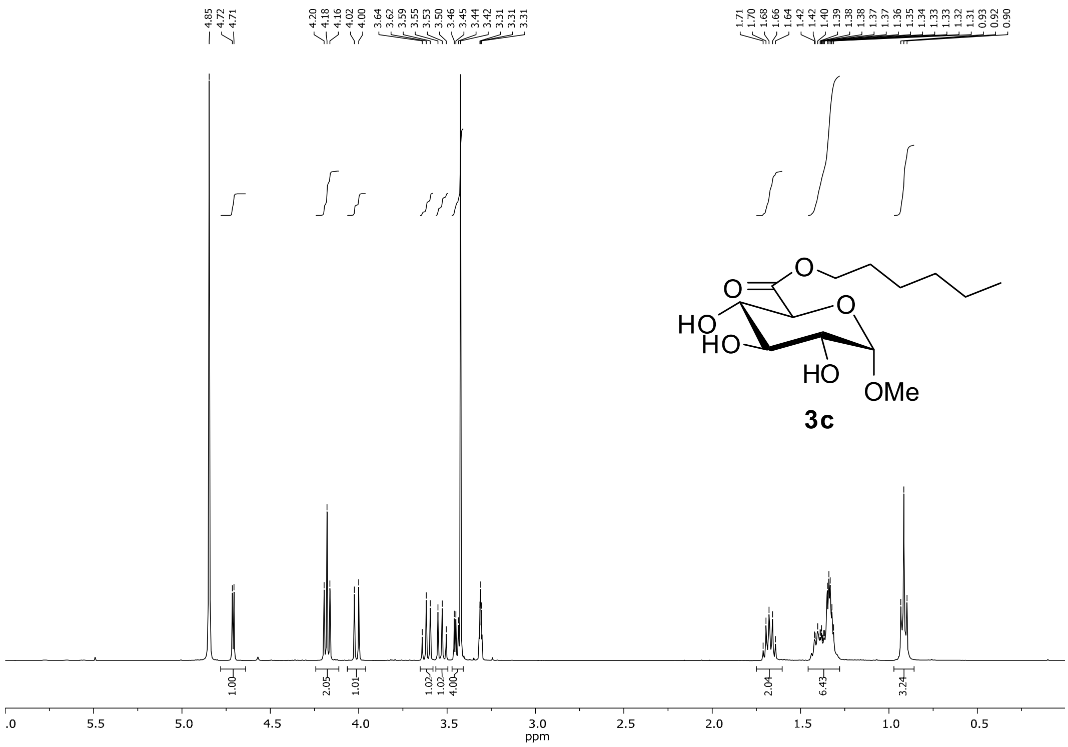

Figure S7. ^{1}H-NMR spectra of hexyl (methyl *α*-D-glucopyranosid)uronate **3c**.

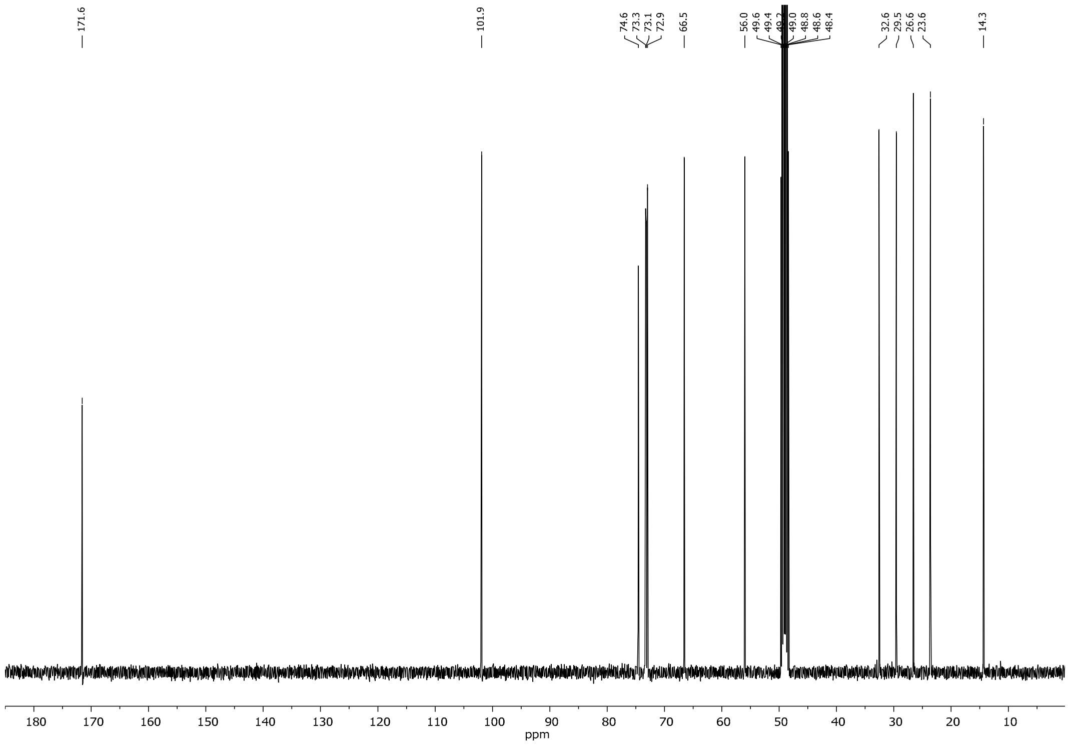

Figure S8. ^{13}C-NMR spectra of hexyl (methyl *α*-D-glucopyranosid)uronate **3c**.

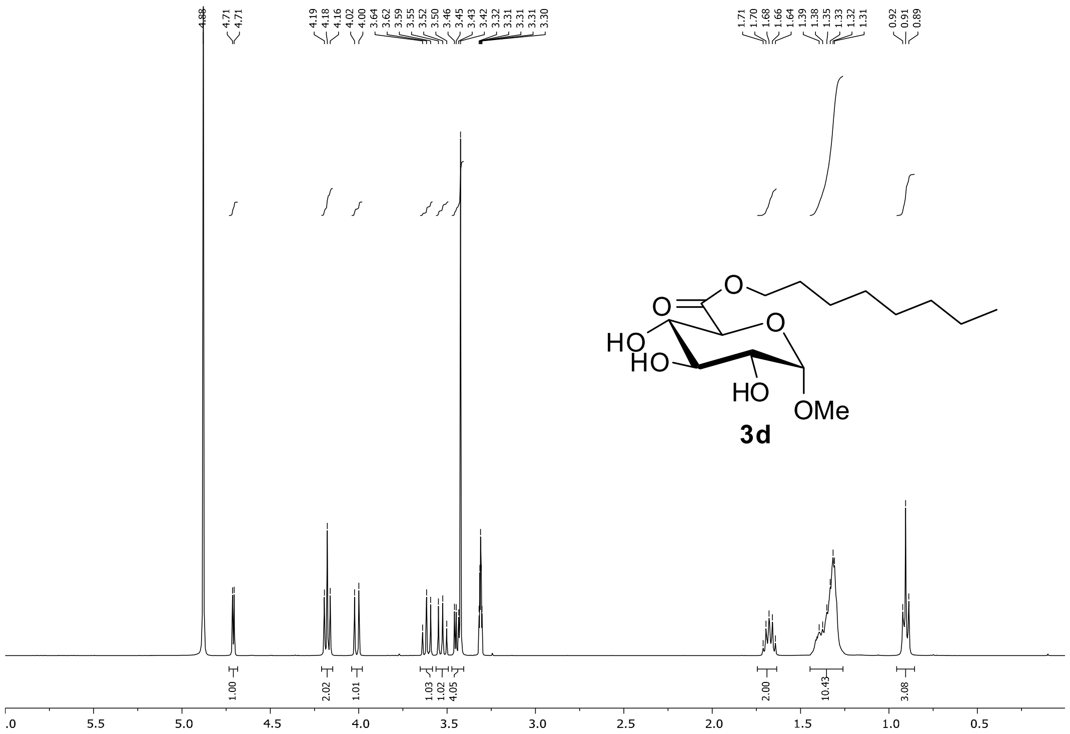

Figure S9. ¹H-NMR spectra of octyl (methyl α-D-glucopyranosid)uronate **3d**.

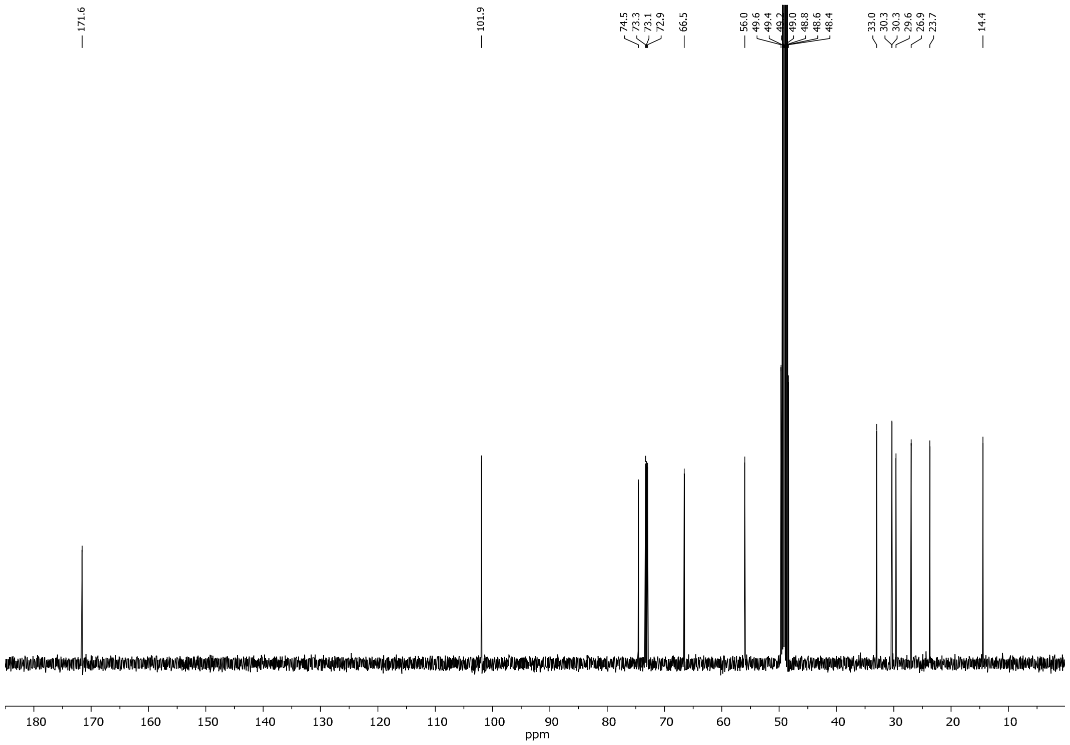

Figure S10. ¹³C-NMR spectra of octyl (methyl α-D-glucopyranosid)uronate **3d**.

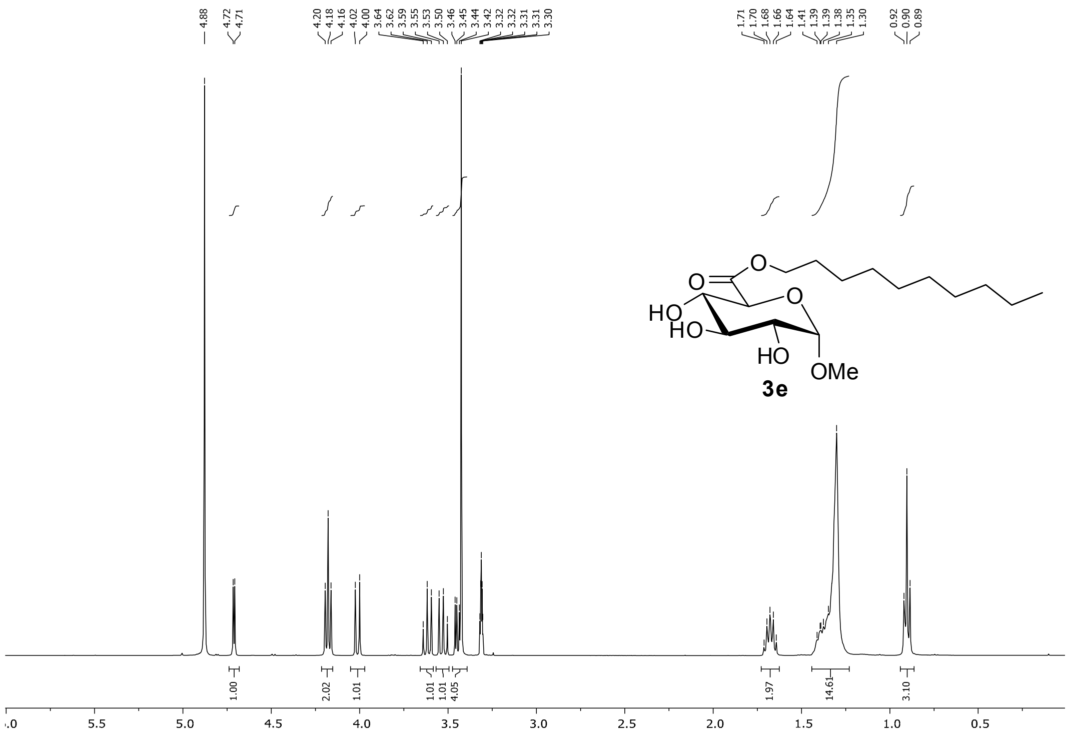

Figure S11. ^{1}H-NMR spectra of decyl (methyl α-D-glucopyranosid)uronate **3e**.

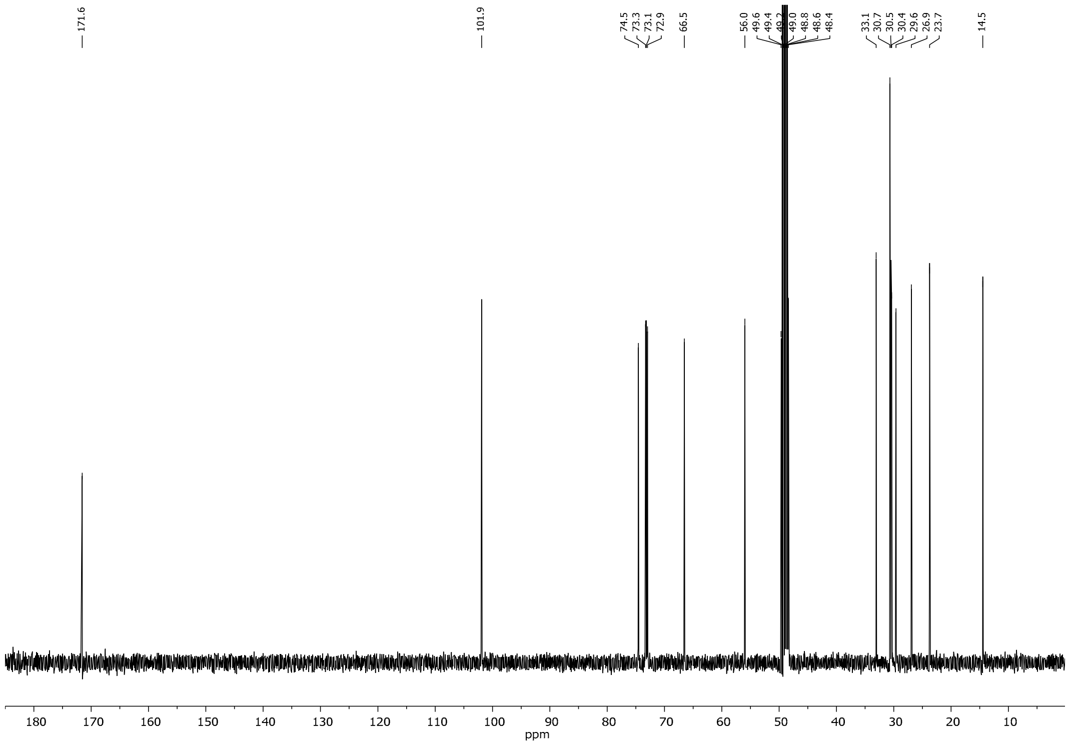

Figure S12. ^{13}C-NMR spectra of decyl (methyl α-D-glucopyranosid)uronate **3e**.

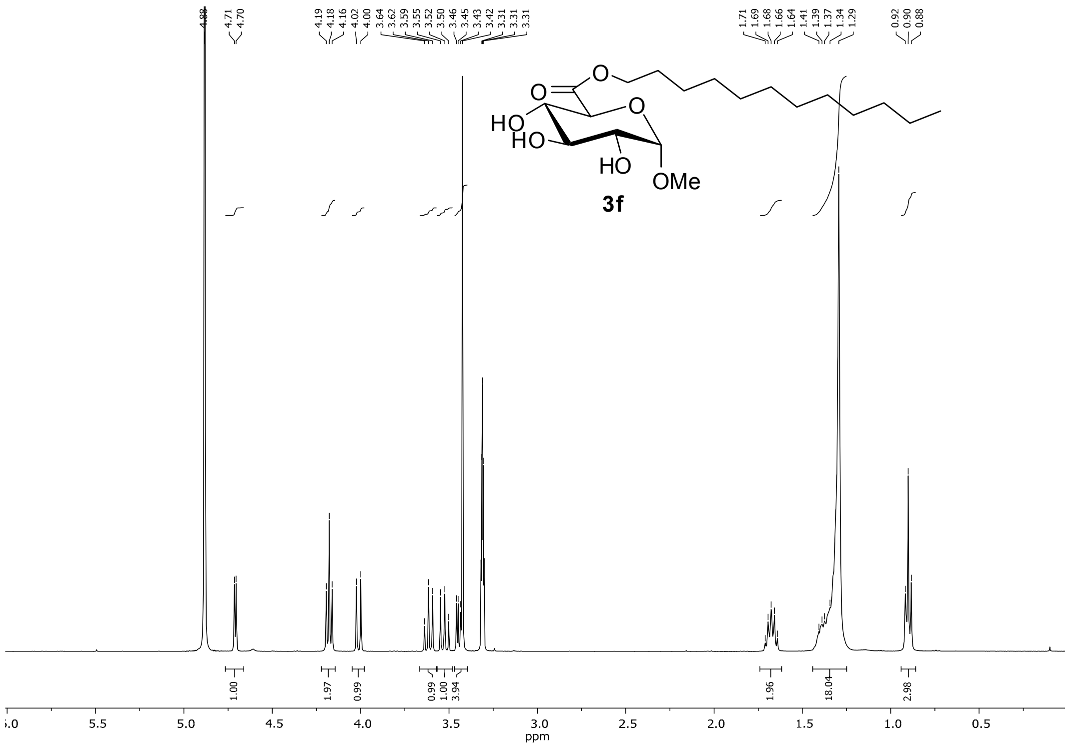

Figure S13. ^{1}H-NMR spectra of dodecyl (methyl α-D-glucopyranosid)uronate **3f**.

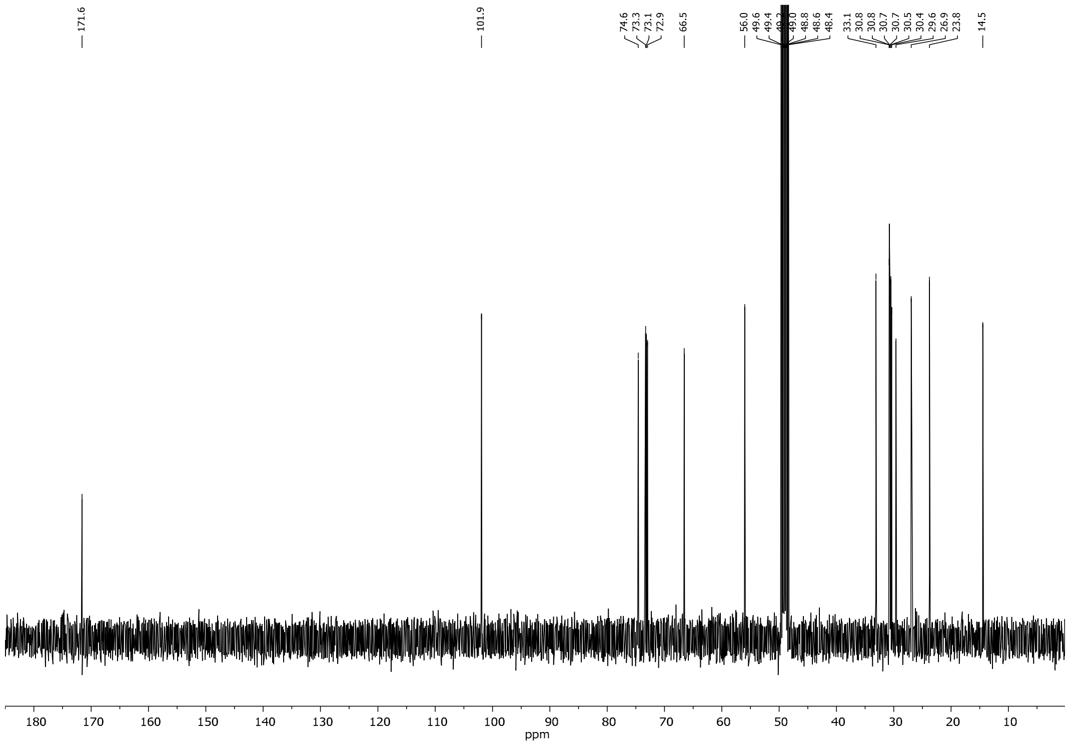

Figure S14. ^{13}C-NMR spectra of dodecyl (methyl α-D-glucopyranosid)uronate **3f**.

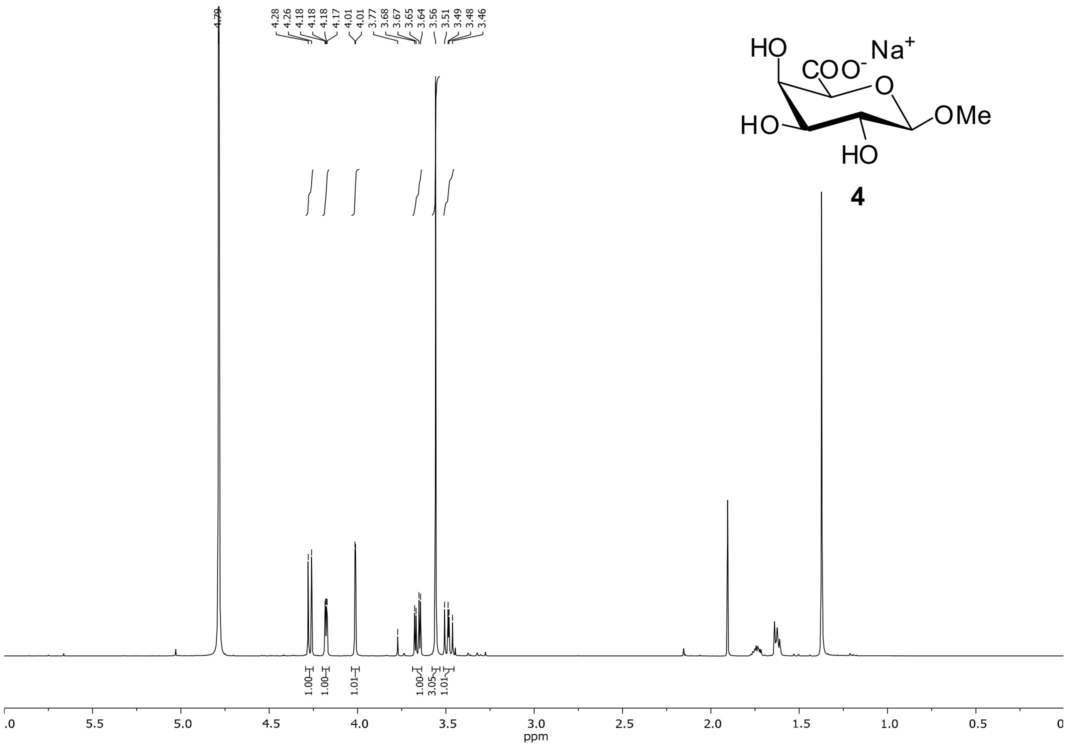

Figure S15. ^{1}H-NMR spectra of sodium (methyl β-D-galactopyranosid)uronate **4**.

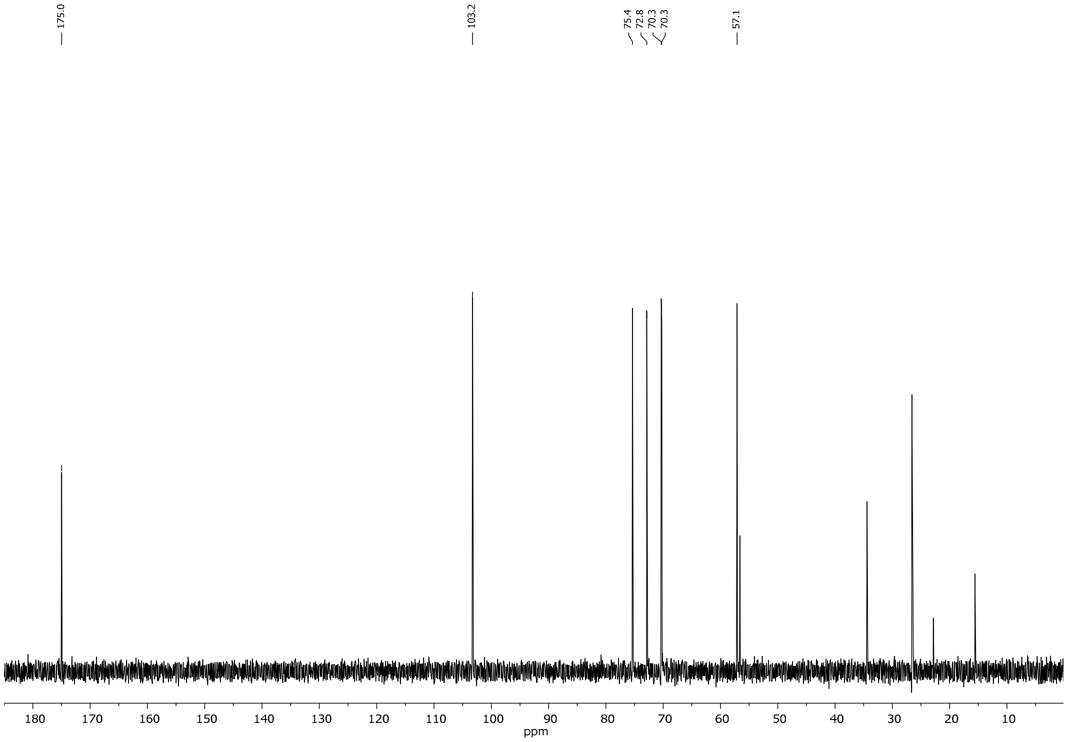

Figure S16. ^{13}C-NMR spectra of sodium (methyl β-D-galactopyranosid)uronate **4**.

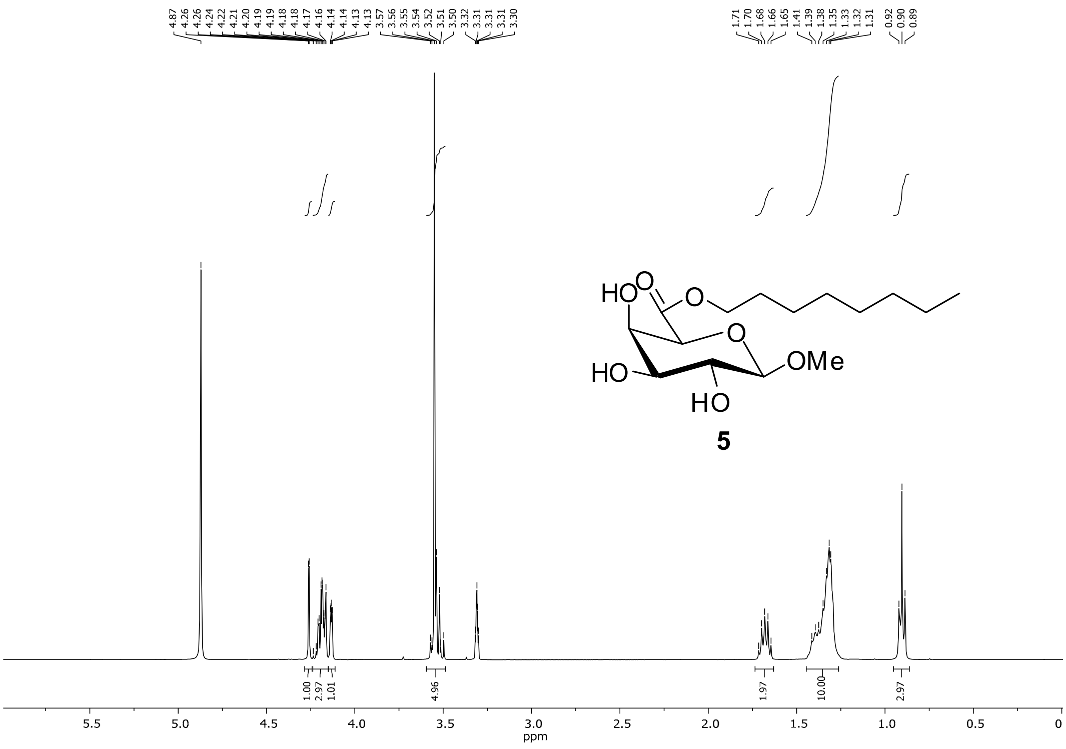

Figure S17. ^{1}H-NMR spectra of octyl (methyl β-D-galactopyranosid)uronate **5**.

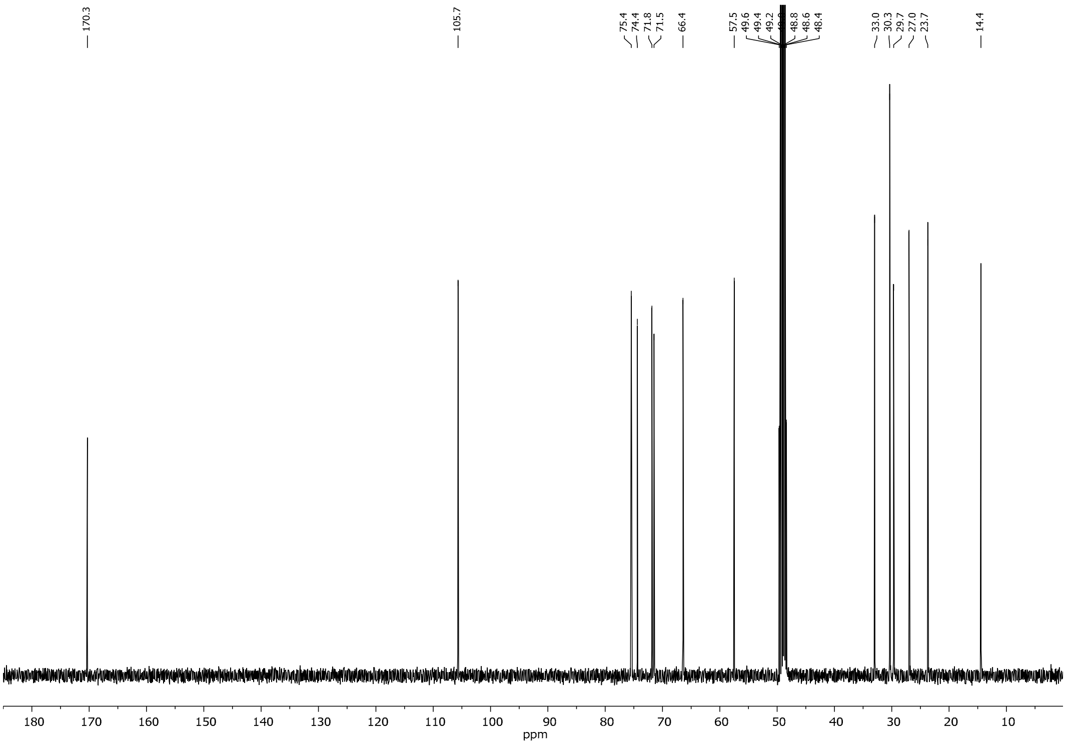

Figure S18. ^{13}C-NMR spectra of octyl (methyl β-D-galactopyranosid)uronate **5**.

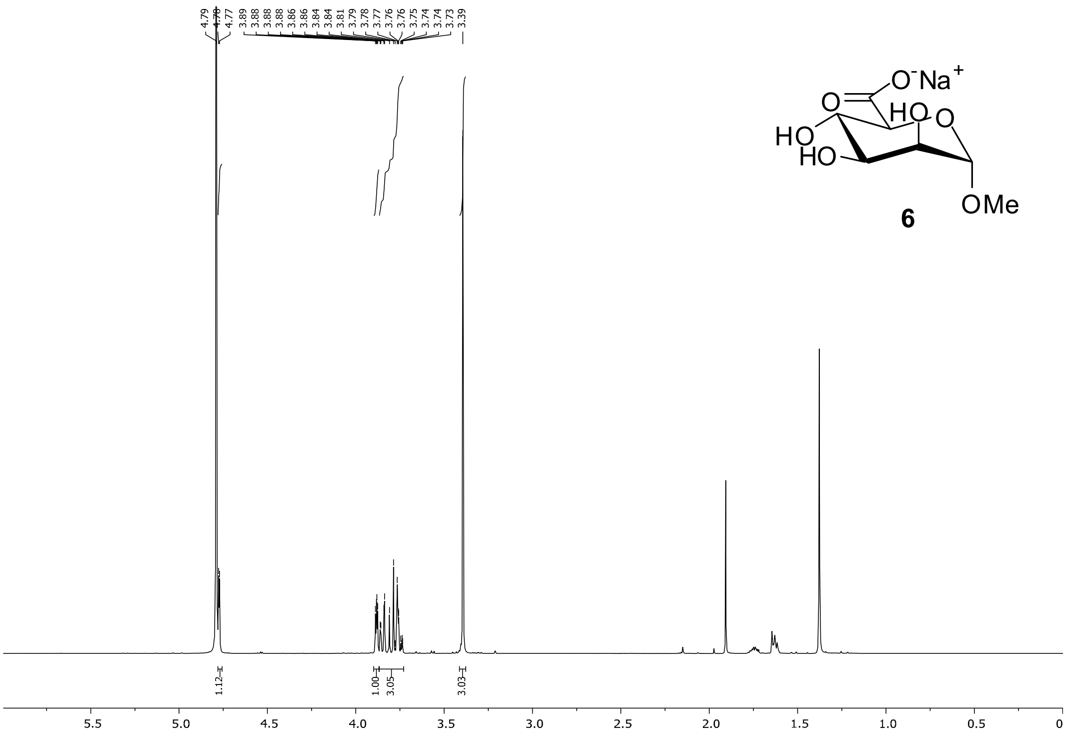

Figure S19. ^{1}H-NMR spectra of sodium (methyl α-D-mannopyranosid)uronate **6**.

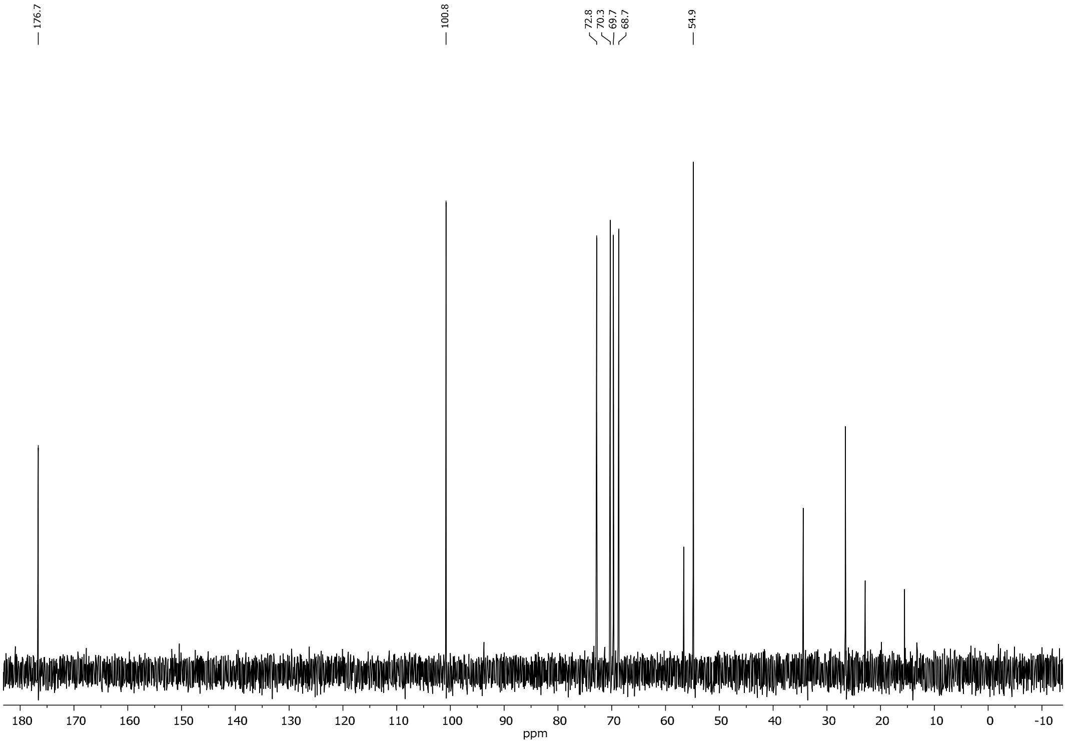

Figure S20. ^{13}C-NMR spectra of sodium (methyl α-D-mannopyranosid)uronate **6**.

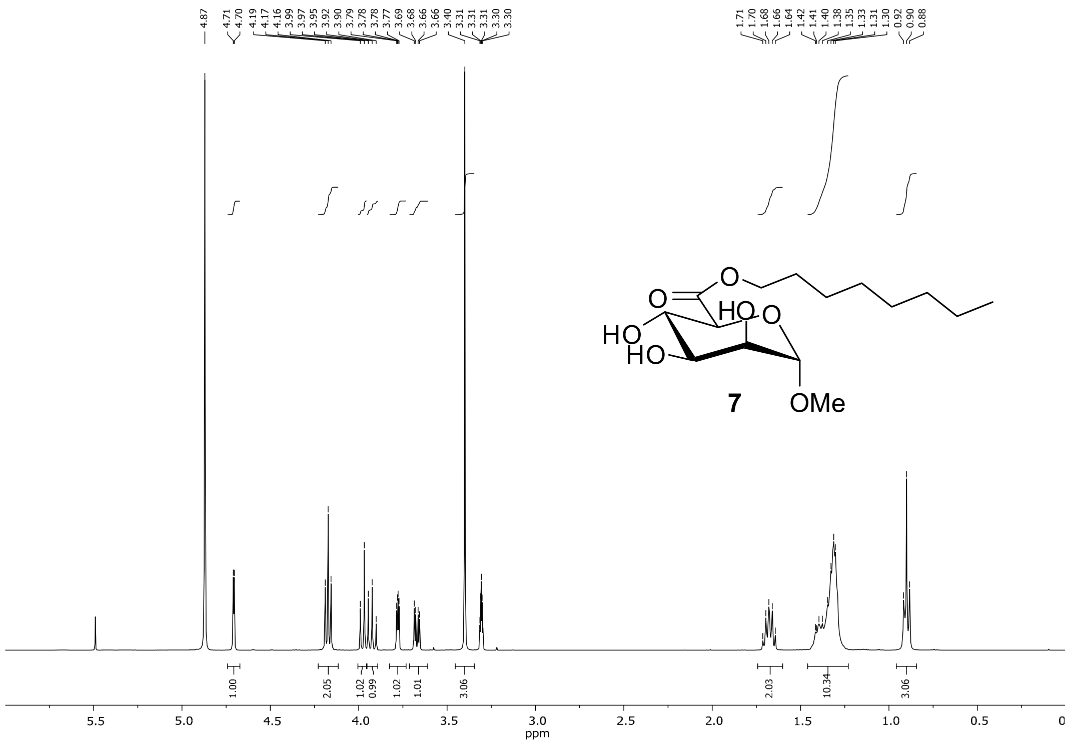

Figure S21. ^{1}H-NMR spectra of octyl (methyl α-D-mannopyranosid)uronate **7**.

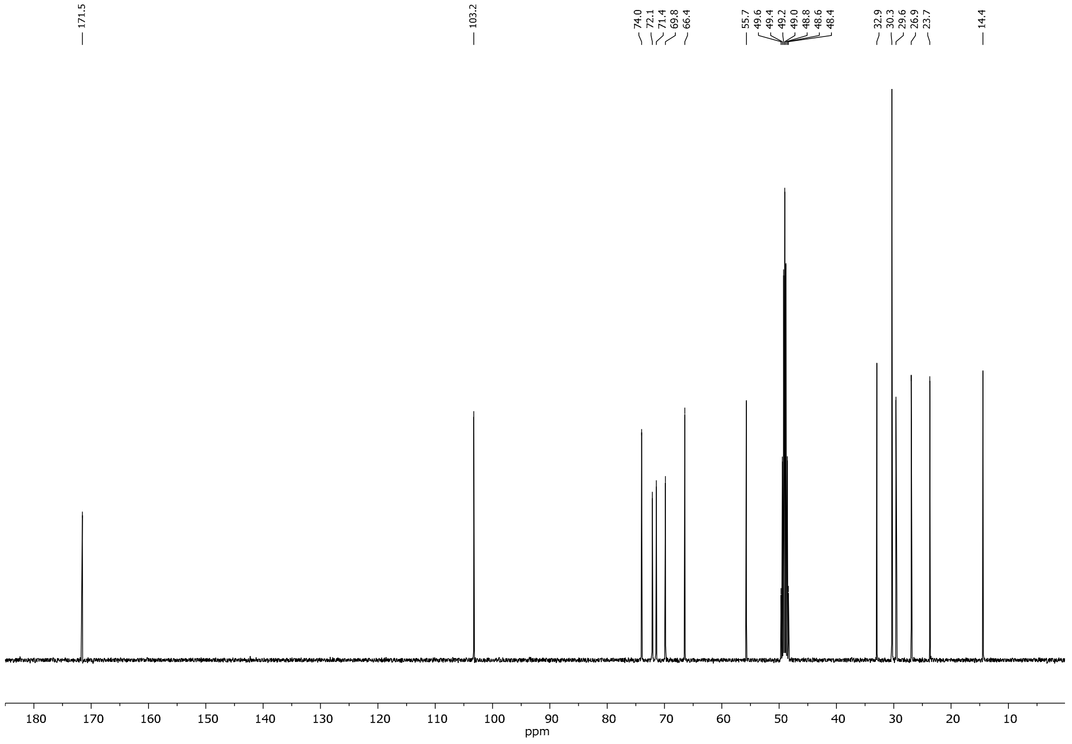

Figure S22. ^{13}C-NMR spectra of octyl (methyl α-D-mannopyranosid)uronate **7**.
